# Supplementary material for: Structural and functional analysis of natural capsid variants suggests sialic acid-independent entry of BK polyomavirus
Source: Cell Rep. 2023 Feb 14;42(2):112114. doi: 10.1016/j.celrep.2023.112114 (PMC9989821; doi:10.1016/j.celrep.2023.112114)
Supplement: Document S1. Tables S1 and S3 and Figures S1 and S2 [file mmc1.pdf]

Cell Reports, Volume *42*

Supplemental information

Structural and functional analysis of natural capsid variants suggests sialic acid-independent entry of BK polyomavirus

Marie N. Sorin, Antonio Di Maio, Lisete M. Silva, Domenic Ebert, Clément P. Delannoy, Ngoc-Khanh Nguyen, Yann Guerardel, Wengang Chai, Franck Halary, Karine Renaudin-Autain, Yan Liu, Céline Bressollette-Bodin, Thilo Stehle, and Dorian McIlroy

**Patient code**	3.4	3.5	3.9
**Age at KTx**	64	27	56
**Sex**	M	M	M
**Graft type**	Kidney 1st graph	Kidney 1st graft	Kidney 2nd graft*
**Donor**	Deceased	Deceased	Living familial donor
**HLA mismatches**	3	4	4
**Immunosuppressive drug regimen**	MMF Tacrolimus	MMF Tacrolimus Corticoids	Azathioprine Tacrolimus
**Log10 BKPyV gIb2 neutralizing titre at KTx**	2.7	3.3	4.6 (Before KTx2) Serum at KTx1 not available
**Log10 BKPyV gIb2 neutralizing titre at M12 post-KTx**	4.1	5.1	4.4 (After KTx2)

*The first graft was kidney and pancreas, and ended with PyVAN, requiring transplantectomy followed by a second kidney graft.

Table S1, Related to Results – Patients clinical data

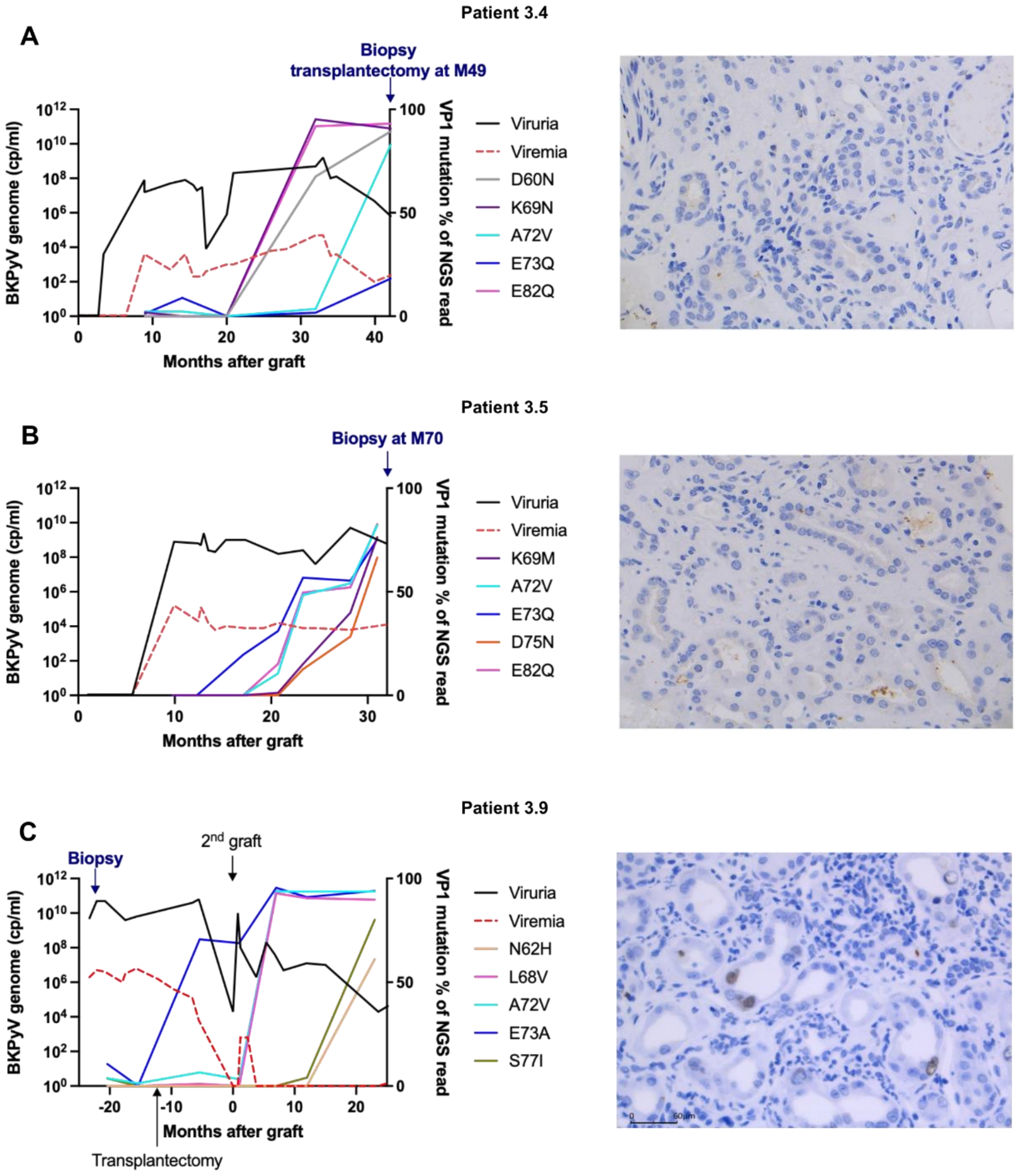

Figure S1, Related to Results. Virological and histological features of BKPyV infection in patients

Left panels: Viruria, DNAemia and VP1 mutation % of NGS reads as a function of time after graft for the three kidney recipients whose VP1 variants were studied. (A) Patient 3.4; (B) Patient 3.5; (C) Patient 3.9. Right panels: SV40 TAg staining of biopsies from patients 3.4 - no significant staining; 3.5 - no significant staining (M70 biopsy is shown); and 3.9 - focal nuclear positivity of tubular epithelial cells. Biopsy images were taken at same magnification. Scale bar is 60 μm.

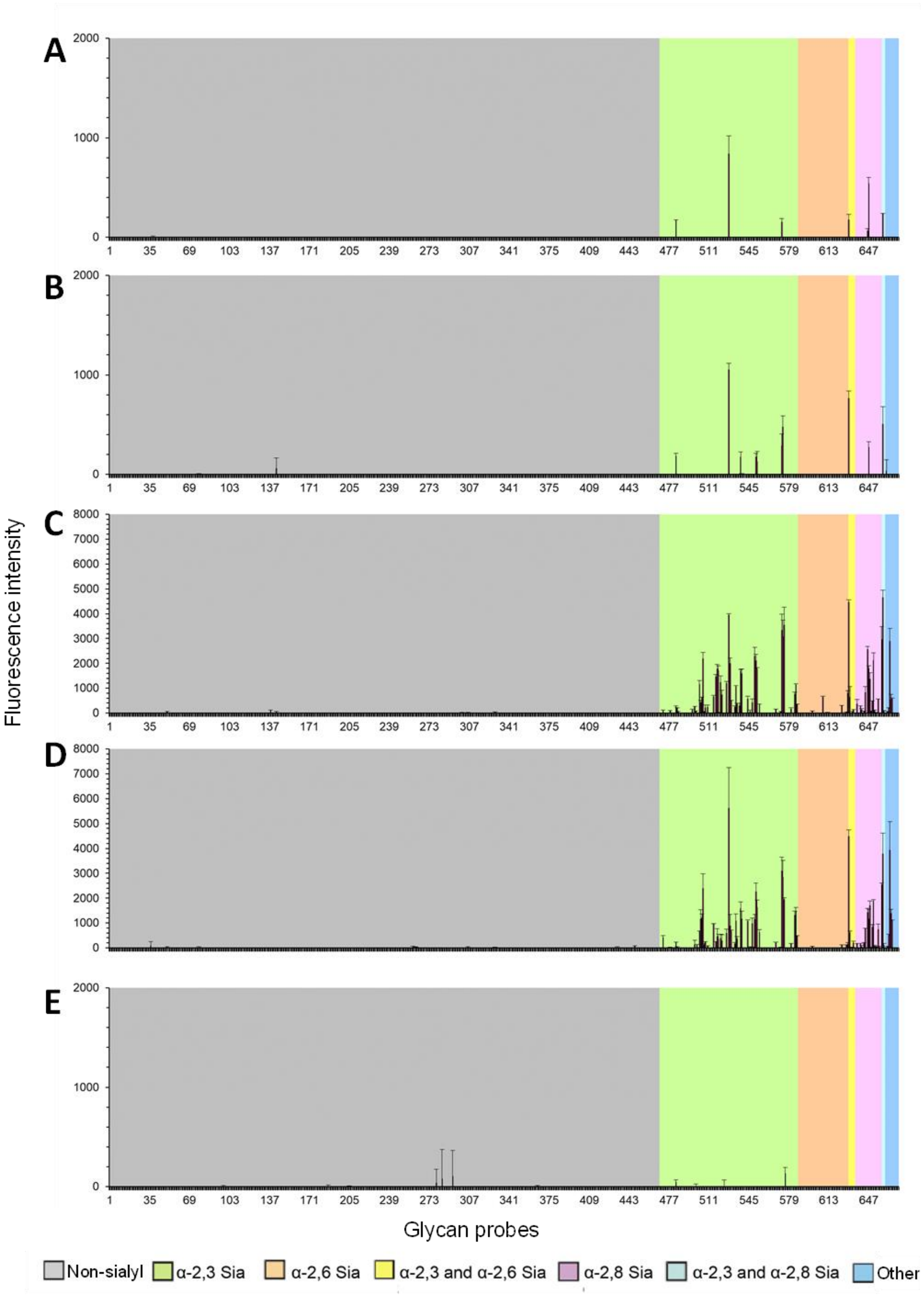

Figure S2, Related to Results. Glycan microarray screening analyses of His-tagged pentameric BKPyV VP1 proteins, wt (A), E73A mutant (B), E73Q mutant (C), VQQ mutant (D) and N-Q mutant (E). The results are the means of fluorescence intensities of duplicate spots, printed at 5 fmol per spot. The error bars represent half of the difference between the two values. In the glycan array the 672 lipid-linked probes are grouped according to sialyl linkages as annotated by the colored panels. The list of glycan probes, their sequences and binding scores are in Table S2.

<table>
<tr><th>Classification</th><th>Guidelines</th></tr>
<tr><td colspan="2">1. Sample: Glycan Binding Sample</td></tr>
<tr><td>Description of Sample</td><td><u>Sample names</u>:
Wild type (WT) BKPyV VP1
Single mutants: E73A BKPyV VP1, E73Q BKPyV VP1
Triple mutant: A72V-E73Q-E82Q (VQQ) BKPyV VP1
Double mutant: K69N-E82Q (N-Q) BKPyV VP1
<u>Origin</u>: recombinant
<u>Method of preparation:</u>
Please see the “Protein expression and purification” section under Materials and Methods in the main text.</td></tr>
<tr><td>Sample modifications</td><td>Not relevant.</td></tr>
<tr><td>Assay protocol</td><td>Microarray analyses were performed essentially as described [S1], for modifications of the protocol please see “Glycan microarray screening” under Materials and Methods section in the main text.</td></tr>
<tr><td colspan="2">2. Glycan Library</td></tr>
<tr><td>Glycan description for defined glycans</td><td>Two glycan microarrays were used, both containing sequence-defined lipid-linked oligosaccharide probes, glycolipids or neoglycolipids (NGLs).
1) The ‘Ganglioside-focused array’ contained 25 ganglioside related probes. These are a sub-set of an array containing 64 glycan probes (in-house designation ‘Neuro-Glycan Array Set 1’, which will be published elsewhere). The names and sequences of the 25 probes are given in Figure 2A.
2) A broad-spectrum screening microarray contained 672 sequence-defined oligosaccharide probes (Table S2). These are a sub-set of a recently generated large screening microarray containing around 900 glycan probes (in-house designation ‘Array Sets 42-56’, which will be published elsewhere).
The NGL probes are from the collection assembled in the course of research in the Glycosciences Laboratory (https://glycosciences.med.ic.ac.uk/glycanLibraryList.html).</td></tr>
<tr><td>Glycan description for undefined glycans</td><td>Not relevant.</td></tr>
<tr><td>Glycan modifications</td><td>No modification was carried out for natural glycolipids.
For NGLs, unless otherwise specified these were prepared from reducing oligosaccharides by reductive amination with the amino lipid, 1,2-dihexadecyl-</td></tr>
</table>

	sn-glycero-3-phosphoethanolamine [(DHPE) [S2]]; AO, NGLs prepared from reducing oligosaccharides by oxime ligation with an aminooxy functionalized DHPE [(AOPE) [S3]].<br>For full description on the definition of lipid moieties of the glycan probes please see https://glycosciences.med.ic.ac.uk/docs/lipids.pdf.
**3. Printing Surface; e.g., Microarray Slide**	
Description of surface	Nitrocellulose-coated glass microarray slides.
Manufacturer	16-pad UniSart® 3D Microarray Slide from Sartorius (Goettingen, Germany)
Custom preparation of surface	Not relevant.
Non-covalent Immobilisation	The lipid-linked oligosaccharide probes were formulated as liposomes by adding carrier lipids, 1,2-dihexanoyl-*sn*-glycero-3-phosphocholine (DHPC) and cholesterol for arraying and non-covalent immobilization on nitrocellulose-coated glass slides [S1].
**4. Arrayer (Printer)**	
Description of Arrayer	Nano-Plotter 2.1 (GeSiM, Radeberg, Germany).
Dispensing mechanism	Non-contact liquid delivery with four dispensing tips.
Glycan deposition	Approximately 0.33 nl was printed per spot.<br>Lipid-linked glycan probes were printed at 2 and 5 fmol per spot in duplicate.
Printing conditions	The printing solutions were all aqueous based. Printing was performed at ambient temperature and relative humidity of 58%.<br>The 'liposome' printing solutions contained 100 pmol/μl of DHPC and cholesterol (both from SIGMA) as lipid carriers in addition to the lipid-linked glycan probes. The concentrations of the lipid-linked glycan probes were 5 and 15 pmol/μl for the 2 and 5 fmol per spot levels, respectively.<br>The printing solutions also contained Cy3 NHS ester (GE Healthcare) at 20 ng/ml (26 fmol/μl) as a marker to monitor the printing process.
**5. Glycan Microarray with "Map"**	
Array layout	The 'Ganglioside-focused array' was printed in 16-pad format. Each array slide contained 16-pad subarrays. Each pad was set up for printing 64 probes maximum, each at 2 levels in duplicate (four spots for one probe in a row); up to 256 spots (16x16) in total in each pad.<br>The 672 lipid-linked probes in the screening arrays were printed on multiple subarrays for parallel binding analyses.

Glycan identification and quality control	The 'Ganglioside-focused array' was analysed with cholera toxin and a number of polyomavirus VP1 proteins with known specificities, e.g. simian virus 40 VP1 [S4] for quality control purposes.<br>The quality control of the screening microarrays of sequence-defined glycan probes was carried out with: biotinylated plant lectins - *Ricinus Communis* Agglutinin I (RCA_{120}), *Aleuria aurantia* lectin (AAL), Concanavalin A (ConA) and WGA (Vector Laboratories), a wide range of anti-carbohydrate antibodies, and a number of viral adhesive proteins that we have published previously.<br>These data will be described elsewhere and are available upon request.
**6. Detector and Data Processing**	
Scanning hardware	GenePix 4300A (Molecular Devices, UK)
Scanner settings	Scanning resolution: 10 µm / pixel<br>Laser channel: Red (scan wavelength 635 nm)<br>PMT: 350<br>Scan power: 100% to achieve maximum signal without spot saturation.
Image analysis software	GenePix® Pro 7 (Molecular Devices)
Data processing	The gpr files were entered into an in-house microarray database using software (designed by Mark Stoll, http://www.beilstein-institut.de/en/publications/proceedings/glyco-2009) for data processing. No particular normalization method or statistical analysis was used for the results of the screening arrays.
**7. Glycan Microarray Data Presentation**	
Data presentation	The microarray binding results are in **Figure 2, Figure S2** and **Table S2.**
**8. Interpretation and Conclusion from Microarray Data**	
Data interpretation	No software or algorithms were used to interpret processed data.
Conclusions	The wild type BKPyV VP1 and its E73A, E73Q and VQQ mutants bound to sialylated glycan probes in the arrays with different binding profiles and fluorescence intensities. No significant binding was detected with the N-Q mutant VP1.

Table s3, Related to STAR*METHODS - Supplementary glycan microarray document based on MIRAGE Glycan Microarray guidelines (doi:10.3762/mirage.3).

References:

[S1] Liu, Y., Childs, R. A., Palma, A. S., Campanero-Rhodes, M. A., Stoll, M. S., Chai, W., & Feizi, T. (2012). Neoglycolipid-based oligosaccharide microarray system: preparation of NGLs and their noncovalent immobilization on nitrocellulose-coated glass slides for microarray analyses. Methods in molecular biology (Clifton, N.J.), 808, 117–136. https://doi.org/10.1007/978-1-61779-373-8_8

[S2] Chai, W., Stoll, M. S., Galustian, C., Lawson, A. M., & Feizi, T. (2003). Neoglycolipid technology: deciphering information content of glycome. Methods in enzymology, 362, 160–195. https://doi.org/10.1016/S0076-6879(03)01012-7

[S3] Liu, Y., Feizi, T., Campanero-Rhodes, M. A., Childs, R. A., Zhang, Y., Mulloy, B., Evans, P. G., Osborn, H. M., Otto, D., Crocker, P. R., & Chai, W. (2007). Neoglycolipid probes prepared via oxime ligation for microarray analysis of oligosaccharide-protein interactions. Chemistry & biology, 14(7), 847–859. https://doi.org/10.1016/j.chembiol.2007.06.009

[S4] Campanero-Rhodes, M. A., Smith, A., Chai, W., Sonnino, S., Mauri, L., Childs, R. A., Zhang, Y., Ewers, H., Helenius, A., Imberty, A., & Feizi, T. (2007). N-glycolyl GM1 ganglioside as a receptor for simian virus 40. Journal of virology, 81(23), 12846–12858. https://doi.org/10.1128/JVI.01311-07
