# Supplementary material for: Structural and functional analysis of natural capsid variants suggests sialic acid-independent entry of BK polyomavirus
Source: Cell Rep. 2023 Feb 14;42(2):112114. doi: 10.1016/j.celrep.2023.112114 (PMC9989821; doi:10.1016/j.celrep.2023.112114)
Supplement: Document S2. Article plus supplemental information [file mmc3.pdf]

Cell Reports

Article

Structural and functional analysis of natural capsid variants suggests sialic acid-independent entry of BK polyomavirus

Graphical abstract

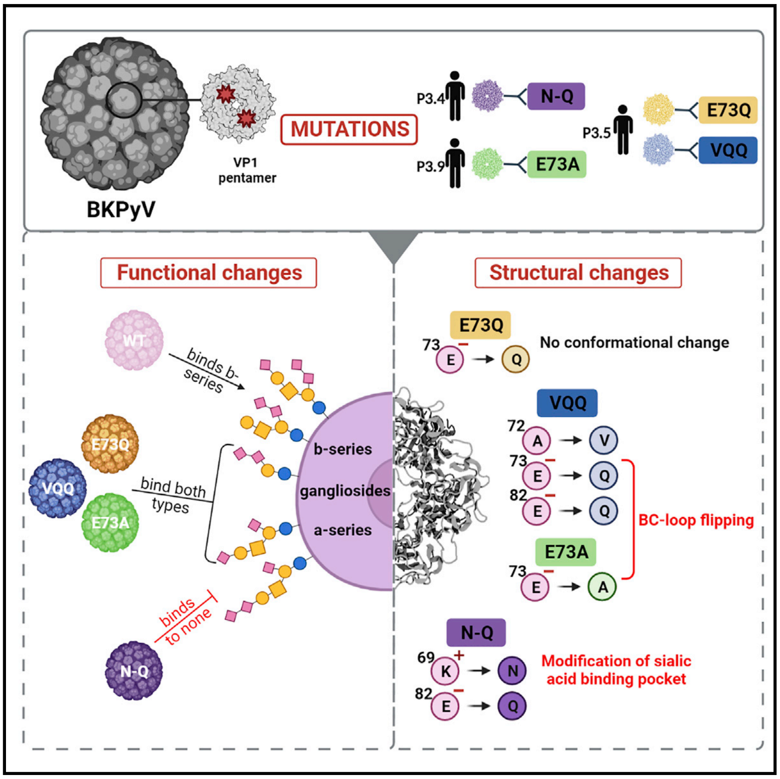

Highlights

- Patient-derived mutations in BKPyV impact host glycan binding specificity
- N-Q variant loses ability to bind sialic acid glycans due to K69N mutation
- VQQ, E73Q, and E73A variants bind to a broader range of gangliosides
- Lower infectivity of VQQ and E73A correlates with altered VP1 BC loop configuration

Authors

Marie N. Sorin, Antonio Di Maio, Lisete M. Silva, ..., Céline Bressollette-Bodin, Thilo Stehle, Dorian McIlroy

Correspondence

dorian.mcilroy@univ-nantes.fr

In brief

Sorin et al. analyze the structure and function of naturally occurring mutations in the VP1 capsid protein of BK polyomavirus, finding two distinct patterns. Several variants show broader ganglioside binding, whereas one particular variant loses binding to sialic acid, yet remains infectious, suggesting the use of a different receptor.

Sorin et al., 2023, Cell Reports *42*, 112114
February 28, 2023 © 2023 The Authors.
https://doi.org/10.1016/j.celrep.2023.112114

CellPress

Cell Reports

CellPress
OPEN ACCESS

Article

Structural and functional analysis of natural capsid variants suggests sialic acid-independent entry of BK polyomavirus

Marie N. Sorin,[1,2,10] Antonio Di Maio,[3] Lisete M. Silva,[3] Domenic Ebert,[2] Clément P. Delannoy,[4] Ngoc-Khanh Nguyen,[1,11] Yann Guerardel,[4,5] Wengang Chai,[3] Franck Halary,[1] Karine Renaudin-Autain,[6] Yan Liu,[3] Céline Bressollette-Bodin,[1,7,8] Thilo Stehle,[2] and Dorian McIlroy[1,9,12,*]

[1]Nantes Université, CHU Nantes, INSERM, Center for Research in Transplantation and Translational Immunology, UMR 1064, 44000 Nantes, France
[2]Interfaculty Institute of Biochemistry, University of Tübingen, Tübingen, Germany
[3]Glycoscience Laboratory, Department of Metabolism, Digestion and Reproduction, Imperial College London, London, UK
[4]Université de Lille, CNRS, UMR 8576 – UGSF - Unité de Glycobiologie Structurale et Fonctionnelle, 59000 Lille, France
[5]Institute for Glyco-core Research (iGCORE), Gifu University, Gifu, Japan
[6]CHU Nantes Service d'Anatomie et Cytologie Pathologique, Nantes, France
[7]CHU Nantes Laboratoire de Virologie, Nantes, France
[8]Faculté de Médecine, Nantes Université, Nantes, France
[9]Faculté des Sciences et des Techniques, Nantes Université, Nantes, France
[10]Present address: Department of Medical Biochemistry and Biophysics, Umeå University, Umeå, Sweden
[11]Present address: CIMI Paris, Sorbonne Université, Paris, France
[12]Lead contact
*Correspondence: dorian.mcilroy@univ-nantes.fr
https://doi.org/10.1016/j.celrep.2023.112114

SUMMARY

BK polyomavirus (BKPyV) is an opportunistic pathogen that uses the b-series gangliosides GD1b and GT1b as entry receptors. Here, we characterize the impact of naturally occurring VP1 mutations on ganglioside binding, VP1 protein structure, and virus tropism. Infectious entry of single mutants E73Q and E73A and the triple mutant A72V-E73Q-E82Q (VQQ) remains sialic acid dependent, and all three variants acquire binding to a-series gangliosides, including GD1a. However, the E73A and VQQ variants lose the ability to infect ganglioside-complemented cells, and this correlates with a clear shift of the BC2 loop in the crystal structures of E73A and VQQ. On the other hand, the K69N mutation in the K69N-E82Q variant leads to a steric clash that precludes sialic acid binding. Nevertheless, this mutant retains significant infectivity in 293TT cells, which is not dependent on heparan sulfate proteoglycans, implying that an unknown sialic acid-independent entry receptor for BKPyV exists.

INTRODUCTION

BK polyomavirus (BKPyV) is a small, non-enveloped, double-stranded DNA virus with an icosahedral capsid formed by 72 capsomers, where a capsomer is an association of a pentamer of the VP1 protein linked internally to a single copy of either the VP2 or the VP3 proteins. BKPyV is known to interact with the urothelium and kidney epithelium through the gangliosides GT1b (NeuAcα2-3Galβ1-3GalNAcβ1-4(NeuAcα2-8NeuAcα2-3) Galβ1-4Glcβ1- Cer) and GD1b (Galβ1-3GalNAcβ1-4(NeuAcα2-8NeuAcα2-3)Galβ1-4Glcβ1-Cer) but also via other b-series gangliosides characterized by their α2-8-linked-disialyl moieties attached to the first galactose from the reducing end,[1–3] which are glycosphingolipids carrying one or multiple sialic acids. The crystal structure of a BKPyV VP1 pentamer in interaction with GD3 tetrasaccharide (NeuAcα2-8NeuAcα2-3Galβ1-4Glc) shows that multiple loops at the surface of the VP1 form a pocket that directly interacts with the α2,8-disialic acid motif of the b-series gangliosides.[3]

BKPyV is an opportunistic virus with a prevalence of 80% in the worldwide population.[4,5] Usually, infections occur asymptomatically during childhood and then lead to latency or persistence in the kidneys. Active viral replication during persistent infection appears to be suppressed by the host, because high viral loads are only observed in immunosuppressive contexts, such as solid organ or hematopoietic stem cell transplants.[6,7] This is a particular issue in the case of kidney transplant, where replication in the engrafted kidney results in the secretion of BKPyV in urine (viruria) and the presence of BKPyV DNA in the blood (DNAemia). These parameters are followed clinically as non-invasive markers to evaluate the state of the infection in the graft. Viruria higher than 10^7 cp/mL and DNAemia greater than 10^4 cp/mL are associated with BKPyV-associated nephropathy (BKPyVAN), which must be confirmed through biopsy.[8,9]

This is an open access article under the CC BY license (http://creativecommons.org/licenses/by/4.0/).

Persistent and uncontrolled BKPyV replication can lead to kidney graft dysfunction and ultimately loss of the graft.[10–12] Usually, the therapeutic strategy is to reestablish the host immune response against BKPyV by modulating the immunosuppressive treatments without endangering the graft.[13] However, for approximately 25% of BKPyVAN patients, high-level BKPyV replication persists[14] despite immunosuppressive treatment modulation, and these are the patients with the highest risk of subsequent graft loss.[15] Persistent high-level BKPyVAN[16] or DNAemia is accompanied by the accumulation of mutations in the VP1 capsid protein, which cluster around the sialic acid binding pocket.[17]

These mutations appear to be caused by viral genome editing by host APOBEC3 enzymes and lead to neutralization escape,[18] a model in which host innate immune responses supply the mutations that are then selected by the adaptive response. In previous work, we found that VP1 mutations also modified the infectivity of pseudotyped particles, suggesting that neutralization escape mutations could also modify BKPyV tropism.[17]

In this study, we focus on four variant forms of the VP1 protein coming from three kidney recipients who experienced persistent BKPyV replication after graft despite immunosuppressive treatment modulation. Viruses sampled sequentially from these patients accumulated multiple mutations in the BC loop region of the VP1 protein, which is involved in the direct interaction of the virus with sialic acids. The BC loop can be divided into two parts: BC1 and BC2 loops, where each part faces in a different direction.[3] Through both functional assays and structural studies, we investigated how these mutations influence both the tropism and the structure of BKPyV, and we were able to reveal the involvement of a sialic acid-independent receptor in BKPyV infection.

RESULTS

VP1 variants show differing infectious profiles compared with the wild-type strain

To investigate the effect of VP1 mutations on BKPyV tropism, we selected BC loop mutations previously identified in three patients from the Nantes University Hospital KTx cohort with persistent DNAemia caused by subtype Ib2 BKPyV[17] (Figure S1 and Table S1). Pseudoviruses carrying the following VP1 variant proteins were generated: the double mutant K69N E82Q (N-Q) from patient 3.4; the E73Q mutant and the triple mutant A72V E73Q E82Q (VQQ) from patient 3.5; and the E73A mutant observed in patient 3.9. Cell lines 293TT and RS were used to test the infectivity of all variant pseudoviruses as well as wild-type (WT) subtype Ib2 pseudovirus. These cell lines were chosen for their kidney origin and their expression of SV40 TAg for amplification of reporter gene expression. In 293TT cells, the E73Q variant was as infectious as the WT, while E73A and N-Q variants showed 3-fold lower infectivity, and VQQ variant was barely infectious (Figure 1A). In RS cells, the VQQ variant had equivalent or slightly higher infectivity than WT, depending on the experiment, while infectivity of the E73A variant was slightly lower than WT. The strongest differences were observed for the N-Q variant, which was almost non-infectious in this cell line, and the E73Q variant, which showed 5- to 10-fold higher infectivity compared with the WT (Figure 1B).

To gain insights into the basis of the different infectious profiles that we observed of WT and variant pseudoviruses, we characterized the ganglioside profiles of the 293TT and RS cell lines by mass spectroscopy following organic extraction of cell membrane components. Analysis was performed by a combination of MALDI-QIT-TOF-MS and MS/MS of permethylated glycosphingolipids to establish the cellular profiles and the sequence of individual components. Both cell lines were shown to contain monosialylated GM2 and GM3 a-series gangliosides along with neutral globosides. In addition, RS cells, but not 293TT cells, specifically expressed b-series disialylated gangliosides GD2 and GD3 carrying α2-8-linked-disialyl epitopes as well as lower proportions of the a-series ganglioside GD1a substituted by a single sialic acid on both internal and external Gal residues (Figure 1C). Finally, RS cells exhibited the minor disialylated Globo-series ganglioside DSGb5 that has been previously identified on normal and malignant kidney and renal tissues.[19,20]

From these observations and knowing that the mutations are found in the BC loop, which is directly involved in ganglioside binding, we sought to characterize their effects on glycan binding specificity.

N-Q variant infection is sialic acid independent

Since polyomaviruses are known to interact with sialylated glycans, we tested whether the infectivity of mutant pseudoviruses was also sialic acid dependent by infecting cells treated with type V neuraminidase, which removes terminal α-2,3- α-2,6-, and α-2,8-linked sialic acid residues (Figures 1D and 1E). VQQ variant infection was only performed in RS cells and N-Q variant only in 293TT because of the weak infectious capacity of each variant for the other cell line. However, E73Q and E73A variant infectivity was tested in both cell lines. In RS cells, WT, E73Q, VQQ, and E73A BKPyV infectivity was significantly decreased by the removal of sialic acid, leading to a remaining infection of around 15% for WT and E73A PSVs, 50% for VQQ, and 40% for E73Q PSVs. In 293TT cells, the ability of WT BKPyV to infect was also impacted by the lack of sialic acid on the cell surface with only around 10% of remaining infection for WT and E73A and around 20% for the E73Q variant. However, the N-Q variant maintained its ability to infect 293TT cells after sialic acid removal, indicating that its infection occurs in a sialic acid-independent manner. Indeed, its infectious ability was even enhanced by sialic acid removal. Thus, another receptor must be involved to support N-Q variant infection in 293TT cells.

Variants have distinct glycan binding profiles compared with WT

The glycan binding profiles of BKPyV variant VP1 pentamers were compared with that of the WT VP1 by microarray analysis using a ganglioside-focused array comprising 25 ganglioside-related probes, glycolipids, and neoglycolipids (NGLs) (Figure 2A). Consistent with the previous data,[3] the WT BKPyV VP1 showed binding restricted to the internally located α2-8-disialyl glycan sequences as in GD1b and GT1b. The VP1s of the E73A, E73Q, and VQQ variants also bound to these but showed a less stringent requirement for the positioning of sialic acids in

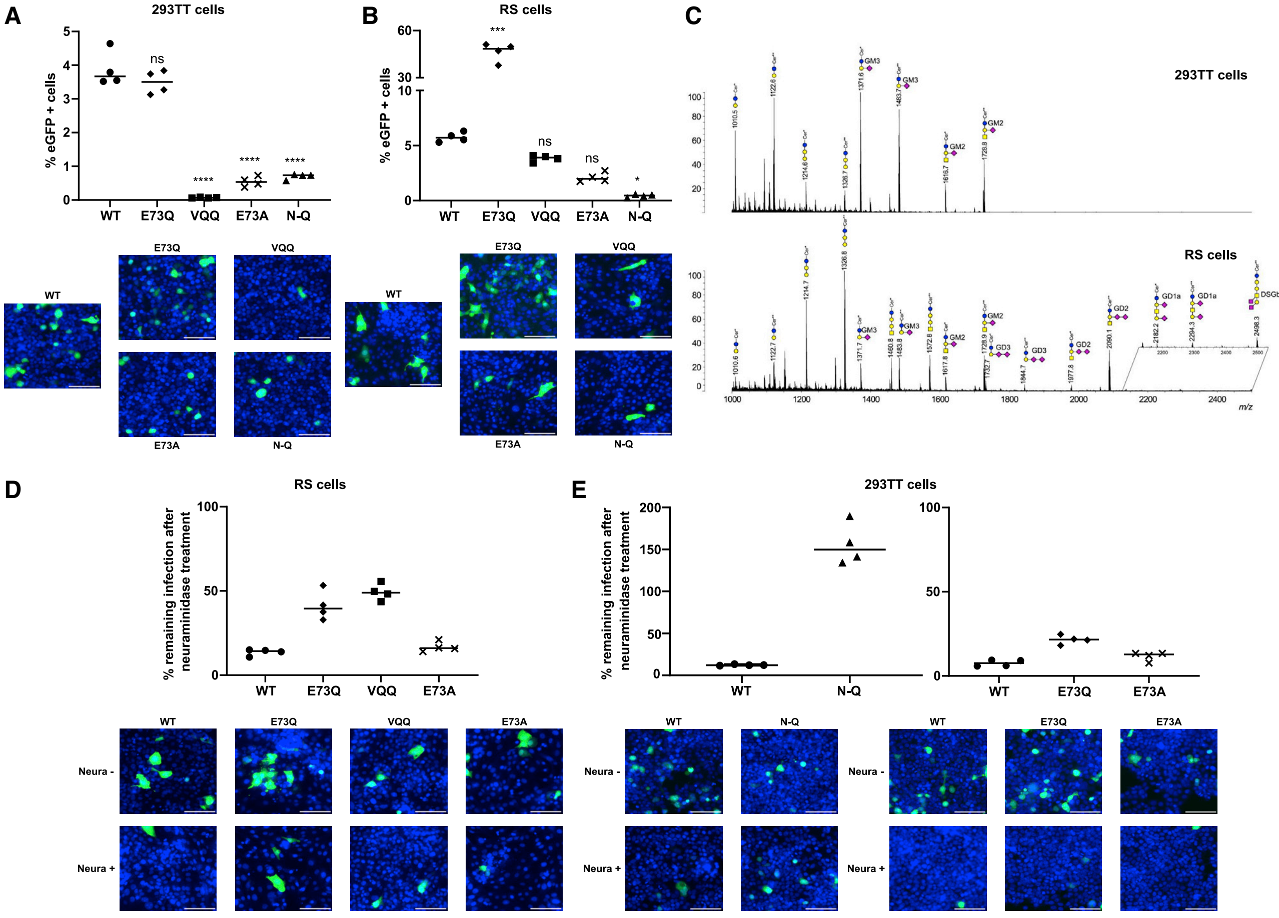

Figure 1. Patient-derived variants of BKPyV have distinct infectious profiles

(A and B) Infectivity assays in 293TT and RS cells. Cells were inoculated with different variants or WT PSVs. Infection was characterized by quantification of GFP^+ cells. Bar corresponds to the median. Each condition was done in quadruplicate. Significant differences were tested through one-way ANOVA followed by Dunnett's test with "WT" condition used as the control group. ****$p < 0.0001$, ***$p < 0.001$, **$p < 0.01$, *$p < 0.05$. Representative images of cells infected by each PSV in both cell lines are associated with each panel. Scale bar: 100 μm.

(C) Comparison of MALDI-TOF-MS profiles of permethylated glycosphingolipids from HEK-293-TT and RS cells. GSLs are present as d18:1/C16:0 (Cer*) and d18:1/C24:0 (Cer**) isomers. yellow circles, galactose; blue circles, glucose; yellow squares, *N*-acetylgalactosamine; purple diamonds, *N*-acetylneuraminic acid.

(D and E) Percentage of remaining infection by PSVs after neuraminidase treatment in 293TT and RS cells. Bar corresponds to the median. Each condition was done in quadruplicate. Representative images of cells with and without neuraminidase infected with different PSVs are associated with each panel. Scale bar: 100 μm.

certain ganglioside structures. In addition to GD1b and GT1b, they showed binding to the ganglio-NGL probes that contain α2-3-linked sialic acid at the non-reducing end galactose as in GM1b-DH and GD1a-DH. The two variants having E to Q mutation at position 73, E73Q and VQQ bound, moreover, to a wider range of gangliosides of both a- and b-series including GD1a, GT1a, GD3, and GQ1b. The overall binding intensities for E73Q and VQQ VP1s were higher compared with the E73A and the WT VP1s. Despite this broader ganglioside binding, none of the VP1 variants bound to GD2, which differs from GD1b only by the absence of the terminal galactose on the non-α2-8-disialyl arm of the glycan. This implies that the galactose residue, which is shared by GD1a, GD1b, GT1a, GT1b, and GQ1b, is likely to contribute to effective ganglioside binding. Indeed, a structural study of whole BKPyV particles complexed with GT1b identified a possible interaction between this galactose and the side chain of the D60 VP1 residue.[21] For the double N-Q mutant, negligible or no significant binding was observed to any of the probes included in the glycan array, indicating the loss of ability to bind to sialylated glycan moieties of gangliosides.

The five VP1 proteins were further analyzed in a broad-spectrum glycan screening array encompassing 672 sequence-defined lipid-linked oligosaccharide probes, representing major types of mammalian glycans found on glycoproteins (*N*-linked and *O*-linked), glycolipids, and proteoglycans, as well as those derived from polysaccharides of bacteria, fungi, and plant origins (Figure S2 and Table S2). The WT and single and triple mutant VP1s (E73A, E73Q, and VQQ) showed binding to sialyl glycans, whereas no binding was observed to the over 400 neutral and sulfated glycan probes that do not contain sialic acids. In overall agreement with findings in the ganglioside-focused arrays, the E73A, E73Q, and VQQ mutants elicited binding to a broader

A

	#	Probe	Structure	WT BKPyV VP1 Score	WT BKPyV VP1 Error	E73A BKPyV VP1 Score	E73A BKPyV VP1 Error	E73Q BKPyV VP1 Score	E73Q BKPyV VP1 Error	VQQ BKPyV VP1 Score	VQQ BKPyV VP1 Error	N-Q BKPyV VP1 Score	N-Q BKPyV VP1 Error
	1	GM4	NeuAcα-3Galβ-Cer	-	1	-	24	101	215	-	14	-	2
o series	2	Asialo-GM2	GalNAcβ-4Galβ-4Glcβ-Cer	-	1	-	10	-	26	-	3	-	4
	3	Asialo-GM1	Galβ-3GalNAcβ-4Galβ-4Glcβ-Cer	-	1	-	3	-	11	-	13	-	13
	4	Asialo-GM1-DH	Galβ-3GalNAcβ-4Galβ-4Glc-DH	-	3	-	59	-	5	-	1	-	6
	5	GM1b	NeuAcα-3Galβ-3GalNAcβ-4Galβ-4Glcβ-Cer	-	8	-	7	-	171	-	22	-	1
	6	GM1b-DH	NeuAcα-3Galβ-3GalNAcβ-4Galβ-4Glc-DH	-	163	1,038	82	1,377	77	1,589	266	-	3
a series	7	GM3	NeuAcα-3Galβ-4Glcβ-Cer	-	-	-	7	-	21	-	16	-	5
	8	GM3(Gc)	NeuGcα-3Galβ-4Glcβ-Cer	-	3	-	7	-	5	-	1	-	12
	9	GM2	GalNAcβ-4Galβ-4Glcβ-Cer<br>NeuAcα-3	-	2	-	10	-	13	-	12	93	72
	10	GM1	Galβ-3GalNAcβ-4Galβ-4Glcβ-Cer<br>NeuAcα-3	-	9	-	4	-	4	-	12	-	-
	11	GM1-DH	Galβ-3GalNAcβ-4Galβ-4Glc-DH<br>NeuAcα-3	-	8	-	6	-	1	-	21	-	1
	12	GM1(Gc)	Galβ-3GalNAcβ-4Galβ-4Glcβ-Cer<br>NeuGcα-3	-	6	-	4	-	18	-	2	-	3
	13	GM1(Gc)-DH	Galβ-3GalNAcβ-4Galβ-4Glc-DH<br>NeuGcα-3	-	2	34	32	446	27	590	236	-	4
	14	GD1a	NeuAcα-3Galβ-3GalNAcβ-4Galβ-4Glcβ-Cer<br>NeuAcα-3	-	4	139	45	415	27	803	129	-	13
	15	GD1a-DH	NeuAcα-3Galβ-3GalNAcβ-4Galβ-4Glc-DH<br>NeuAcα-3	-	2	750	132	1,739	185	2,809	459	-	2
	16	GT1a	NeuAcα-8NeuAcα-3Galβ-3GalNAcβ-4Galβ-4Glcβ-Cer<br>NeuAcα-3	-	2	352	113	1,263	123	1,362	208	-	2
	17	GalNAc-GD1a (Ac,Gc)	GalNAcβ-4Galβ-3GalNAcβ-4Galβ-4Glcβ-Cer<br>NeuAc/NeuGcα-3 NeuGc/NeuAcα-3	-	1	-	56	-	4	-	5	20	6
b series	18	GD3	NeuAcα-8NeuAcα-3Galβ-4Glcβ-Cer	-	7	-	18	69	30	414	56	-	6
	19	GD3-DH	NeuAcα-8NeuAcα-3Galβ-4Glc-DH	-	4	-	21	271	33	706	86	-	24
	20	GD2	GalNAcβ-4Galβ-4Glcβ-Cer<br>NeuAcα-8NeuAcα-3	-	1	-	6	-	3	-	6	-	4
	21	GD1b	Galβ-3GalNAcβ-4Galβ-4Glcβ-Cer<br>NeuAcα-8NeuAcα-3	652	52	1,065	161	2,460	128	2,375	50	-	12
	22	GD1b-DH	Galβ-3GalNAcβ-4Galβ-4Glc-DH<br>NeuAcα-8NeuAcα-3	796	140	1,633	387	3,665	824	3,013	73	-	4
	23	GT1b	NeuAcα-3Galβ-3GalNAcβ-4Galβ-4Glcβ-Cer<br>NeuAcα-8NeuAcα-3	502	36	1,794	264	1,846	95	2,542	447	-	3
	24	GQ1b	NeuAcα-8NeuAcα-3Galβ-3GalNAcβ-4Galβ-4Glcβ-Cer<br>NeuAcα-8NeuAcα-3	105	89	291	62	622	34	1,000	120	-	2
c series	25	GT1c-DH	Galβ-3GalNAcβ-4Galβ-4Glc-DH<br>NeuAcα-8NeuAcα-8NeuAcα-3	42	41	-	42	59	45	-	7	-	-

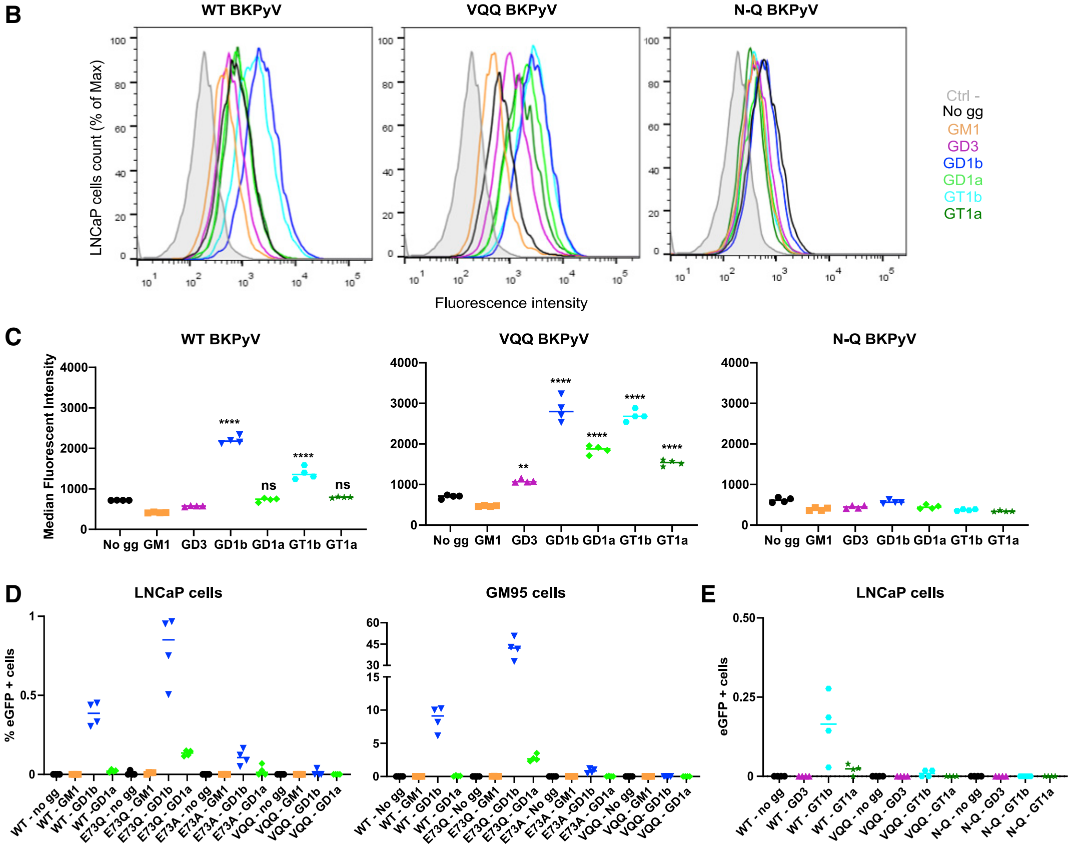

(legend on next page)

range of ganglioside sequences compared with the WT VP1. With the repertoire of sialyl glycans included in the screening arrays, binding to sialyl glycans beyond ganglioside sequences was also observed; among the most strongly bound non-ganglioside glycans are the α2-3-linked sialyl sequence with type 1 *N*-acetyllactosamine (LacNAc) backbone (Galβ1-3GlcNAc-) as in LSTa (position 528, Table S2) and its disialyl analog DSLNT with an internal α2-6-linked sialic acid (position 630). The E73Q and VQQ VP1s showed binding also to several α2-3-sialyl glycans with type 2 LacNAc backbones (Galβ1-4GlcNAc-) with or without fucosylation on GlcNAc and to a number of α2-9-linked polysialic acid sequences (Table S2). It should be noted that the sialyl glycan binding by these two mutant VP1 remains relatively selective, mainly to glycans carrying unmodified *N*-acetylneuraminic acid (NeuAc) on certain glycan backbone sequences and lipid moieties. This highlights the importance of the sialic acid form and glycan presentation for BKPyV VP1 recognition. Here also with the double mutant VP1, N-Q, in sharp contrast to the other four VP1 proteins, binding was not detected to any sialylated or non-sialylated glycans included in the screening arrays. It remains possible that carbohydrate ligands for the N-Q mutant exist but were not included in the probe library included here.

To corroborate the glycan array results, cellular binding assays were performed on LNCaP cells supplemented with a panel of gangliosides (GM1, GD3, GD1b, GT1b, GD1a, and GT1a) with fluorescent WT, VQQ, and N-Q virus-like particles (VLPs) (Figures 2B and 2C). WT VLPs bound to cells supplemented with GD1b and GT1b, consistent with the known role of these gangliosides as BKPyV receptors. VQQ VLPs were able to bind all gangliosides tested except GM1, confirming the glycan array data. Stronger binding was seen for both GD1b and GT1b followed by GD1a and GT1a and then weak binding to cells supplemented with GD3 (Figures 2B and 2C). Also consistent with the glycan array results, N-Q VLPs did not bind any of the tested gangliosides (Figures 2B and 2C).

Gangliosides are not sufficient to support VQQ variant infection

To determine whether binding of the different gangliosides by WT, VQQ, E73Q, and E73A variant VP1 can lead to infection, we performed infectivity assays on LNCaP and GM95 cells supplemented with gangliosides. As expected, infection was seen for WT in cells supplemented with GD1b, but also for E73Q and E73A PSVs under the same condition, and infection was also seen for E73Q when cells were supplemented with GD1a in both LNCaP and GM95 cells (Figure 2D). The ability of the E73Q variant to infect cells supplemented with GD1a was consistent with its increased infectivity in RS cells, which carried GD1a gangliosides. Surprisingly, no infection was observed for VQQ in any supplementation condition, despite its ability to bind GD1b, GD1a, and other gangliosides on the surface of cells. Hence, ganglioside binding was not sufficient for infection with VQQ variant PSV. Finally, as predicted, no infection was seen in any supplementation condition for the N-Q variant, confirming that gangliosides are not used by this variant for infectious entry (Figure 2E).

Mutations at VP1 positions 72 and 73 can lead to BC loop flipping

To understand the impact of the mutations on the VP1 protein structure, variant structures were solved through X-ray crystallography. The single mutants E73Q and E73A and triple mutant VQQ VP1 pentamer structures were solved at 1.85, 1.89, and 1.8 Å (Table 1). All variant VP1 pentamer structures were globally similar to the WT VP1 pentamer with a doughnut-shaped ring composed of a central pore surrounded by five VP1 monomers in 5-fold symmetry. Like the WT structure, the monomers have a β-sandwich fold with jelly roll topology. The eight β-strands (B, I, D, G and C, H, E, F) are linked by extensive loops, exposed at the surface of the protein (Figures 3A and 3B).

The E73Q pentamer structure was almost identical to that of the WT (RMSD in the BC loop region, from amino acids 68–76, of 0.5 Å), whereas the E73A mutant presenting an alanine in the same position showed some structural changes. Specifically, the S-shaped curve of the backbone structure between amino acids 72 to 75 of the BC2 loop appeared to be flipped over in two monomers out of the five (Figure 3C). We therefore refer to this conformational change as "BC loop flipping." However, two monomers retained the WT loop conformation, while the last monomer seemed to have a state where both conformations are superimposed, leading to two different RMSD values for the BC loop of the E73A crystal (1.2 Å for WT loop conformation and 1.5 Å for the alternative conformation). Thus, the mutation to alanine induces a more flexible conformation for the loop compared with the WT and the E73Q single mutant. Interestingly, while the E73Q mutation on its own did not induce any structural changes, when combined with A72V, as seen in the VQQ variant, the second part of the BC loop was switched (RMSD of 2.8Å) in a similar manner to that seen in the E73A variant (Figure 3C). However, in the case of the VQQ variant,

Figure 2. BKPyV variants have distinct glycan binding profiles

(A) Results table showing the ganglioside-focused glycan microarray data including the list of glycan probes, their sequences and lipid moieties, their fluorescence binding scores, and relative binding intensities ("heatmap") elicited with the His-tagged BKPyV VP1s. Lipid moieties: Cer, ceramide; DH, 1,2-dihexadecyl-*sn*-glycero-3-phosphoethanolamine (DHPE). Fluorescence binding signals are shown as means of duplicate spots at 5 fmol per spot; "–" indicates fluorescence intensity less than 1. Errors represent half of the difference between the two values. Color patterns for relative binding intensity: blue, <10%; yellow, 10%–30%; orange, 30%–70%; red, 70%–100%. 100%, the maximum binding score observed across the five experiments.

(B) Histograms representing fluorescent intensities of Alexa 647 variant or WT VLPs during cell-binding assays on ganglioside-supplemented LNCaP cells. LNCaP cells alone are shown in a filled gray histogram for negative control, and other conditions with VLPs are represented by colored histograms.

(C) Median fluorescence intensity comparison for binding of each VLP to LNCaP cells supplemented with different gangliosides. Bar corresponds to the median. Each condition was done in quadruplicate. Significant differences were tested through one-way ANOVA followed by Dunnett's test with "no gg" condition used as the control group. ****$p < 0.0001$, ***$p < 0.001$, **$p < 0.01$, *$p < 0.05$.

(D and E) Infectivity assays on ganglioside-supplemented LNCaP or GM95 cells with WT, E73Q, E73A, and VQQ PSV (D) and WT, VQQ, and N-Q PSV (E). Infection was characterized through quantification of GFP^+ cells. Bar corresponds to the median. Each condition was done in quadruplicate.

Table 1. Data processing and refinement

Data collection	GI E73Q	GI E73A	GI A72V E73Q E82Q	GI K69N E82Q
PDB ID	8AGO	8AGH	8AH0	8AH1
Space group	P2(1)2(1)2	P2(1)2(1)2	P2(1)2(1)2	P2(1)
Unit cell length (Å)	a = 145.3	a = 144.5	a = 144.7	a = 60.5
	b = 153.0	b = 152.3	b = 152.6	b = 156.0
	c = 62.8	c = 62.6	c = 62.8	c = 141.3
Unit cell angle (°)	α = 90	α = 90	α = 90	α = 90
	β = 90	β = 90	β = 90	β = 92.64
	γ = 90	γ = 90	γ = 90	γ = 90
Pentamers/ASU	1	1	1	2
Resolution (Å)	50–1.85 (1.92–1.85)	50–1.89 (1.96–1.89)	50–1.80 (1.86–1.80)	50–2.01 (2.08–2.01)
Total reflections	1,582,082 (153,046)	1,488,272 (149,590)	1,743,972 (175,371)	1,086,928 (100,014)
Unique reflections	119,532 (11,509)	111,193 (10,995)	129,677 (12,697)	164,438 (17,100)
Rmeas (%)	17.3 (190.2)	13.2 (164.2)	14.7 (168.2)	20.8 (149.2)
Completeness (%)	99.6 (97.4)	99.9 (99.9)	99.9 (99.4)	94.8 (98.73)
CC1/2 (%)	99.9 (73.7)	99.9 (62.6)	99.9 (57.6)	99.4 (57.0)
I/σ	14.0 (1.4)	15.6 (1.7)	13.4 (1.5)	8.4 (1.3)
**Refinement**				
Rwork (%)	19.02	17.44	17.16	18.00
Rfree (%)	22.90	21.13	20.97	23.21
Average B factor (Å^2)	31.0	33.0	29.0	30.0
**RMS deviation**				
Bond length (Å)	0.010	0.010	0.011	0.010
Bond angle (°)	1.60	1.62	1.65	1.71
Wilson B factor (Å^2)	25.8	28.6	24.6	25.9

*Rfree was calculated with 5% of the data $R_{meas} = \frac{\sum hkl \sum hkl \sqrt{\frac{n}{n-1}} \sum_{j=1}^{n} |I_{hkl,j} - \langle I_{hkl} \rangle|}{\sum hkl \sum_{j} I_{hkl,j}}$ $CC_{1/2} = \frac{\sigma_{\tau}^2}{\sigma_{\tau}^2 + \sigma_{\epsilon}^2} = \frac{\sigma_y^2 - \frac{1}{2}\sigma_{\epsilon}^2}{\sigma_y^2 + \frac{1}{2}\sigma_{\epsilon}^2}$ $R = \frac{\sum ||F_{obs}| - |F_{calc}||}{\sum |F_{obs}|}$

Values in parentheses are for the highest-resolution shell.

the modified orientation of the BC loop was observed in all five VP1 monomers, not just in the two monomers seen in E73A. This suggests that mutations at position 73 on their own may not be enough to induce a clear structural change in the loop orientation, which only occurs if mutations in this position are combined with the A72V mutation at the neighboring amino acid.

However, structural changes seen in the VQQ variant are not directly involved in the ganglioside interaction. As previously described, the double sialic part of the b-series gangliosides interacts with the BC1 loop, not the BC2 loop, as well as HI and DE loops.[3] Thus, the structure of the VQQ variant is consistent with its ability to bind gangliosides.

K69N mutation in N-Q variant induces loss of interaction with sialic acids

The structure of the N-Q variant VP1 pentamer was also solved at 2.01 Å (Table 1). Like the other variants, the N-Q VP1 pentamer structure was very similar to the WT VP1 pentamer (Figures 4A and 4B). Although no major change was observed in the backbone orientation of the BC loop that carried the K69N and E82Q mutations, the mutation from lysine to asparagine at position 69 modified the sialic acid binding pocket of the VP1 pentamer (Figure 4C). Lysine 69 is involved directly with the double sialic acid part of the b-series gangliosides by making van der Waals, salt bridge, and hydrogen bond interactions through its side chain, and its replacement by asparagine leads to loss of those interactions (Figure 4D). Moreover, if gangliosides were to bind the pentamer in the same way, the asparagine side chain would be orientated too close (1.3 Å) to the hydroxyl group on C8 of the second sialic acid, leading to a steric clash between the sialic acid and the asparagine.

WT, VQQ, and N-Q BKPyV do not use GAGs for infectious entry

Despite losing its ability to bind sialic acid, the N-Q variant remained infectious in the 293TT cell line, indicating that another receptor is used by this variant to enter these cells. Merkel cell polyomavirus (MCPyV) is known to interact with both sialylated and non-sialylated glycans for infection. Like BKPyV, MCPyV can interact with gangliosides as attachment receptor, but also heparan sulfate (HS) or chondroitin sulfate (CS) glycosaminoglycans (GAGs) as entry receptor.[23] Furthermore, JCPyV and BKPyV PSVs capsid variants that cannot bind sialic acid have also been reported to use GAGs for infectious entry.[21,24] To test whether the BKPyV N-Q variant could interact with GAGs as an alternative

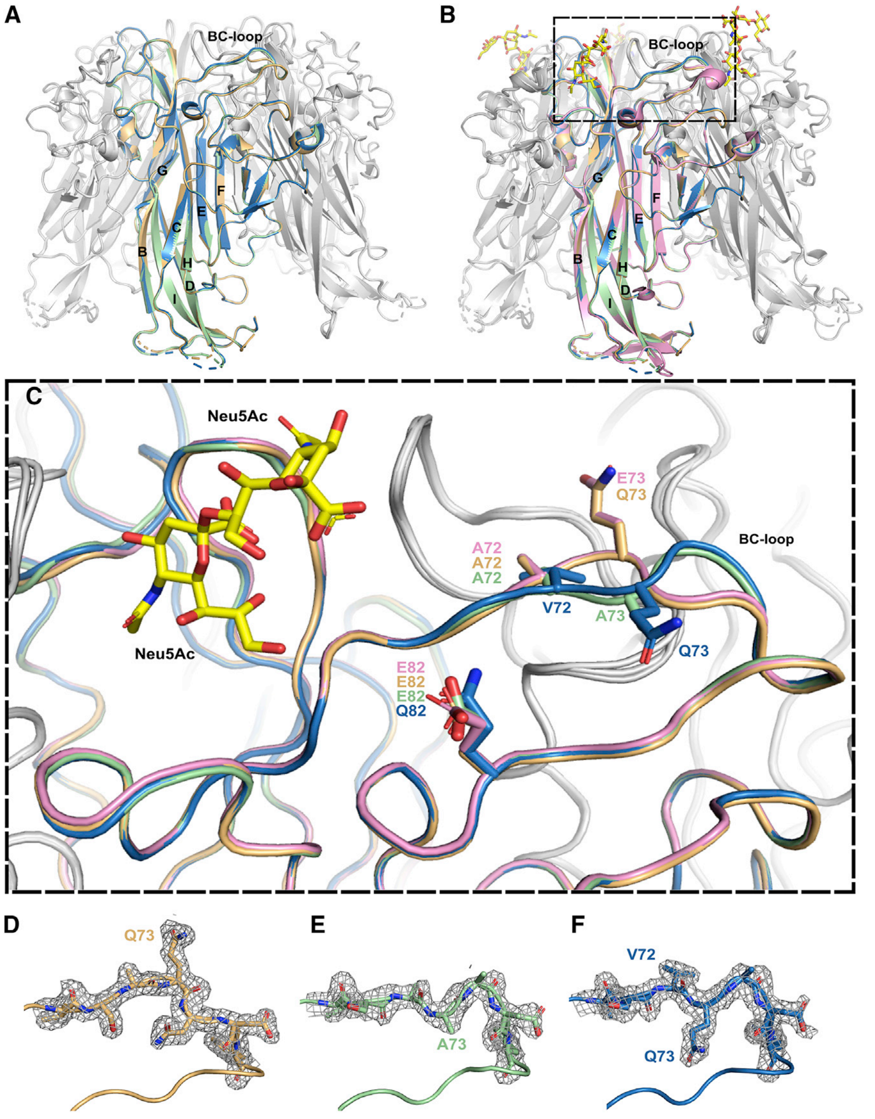

Figure 3. Mutations of amino acids 72 and 73 lead to structural changes in the BC loop conformation

(A) Superposition of variant E73Q (PDB: 8AGO), E73A (PDB: 8AGH), and VQQ (PDB: 8AH0) VP1 pentamer structures. One monomer for each structure is highlighted in orange for E73Q, green for E73A, and blue for VQQ.

(B) Superposition of variant VP1 pentamers with BKPyV WT VP1 pentamer in association with GD3 oligosaccharides (PDB: 4MJ0). WT VP1 monomer is highlighted in pink, and GD3 molecules are represented by yellow sticks.

(C) Zoom on the BC loops of highlighted VP1 monomers. Amino acids 72, 73, and 82 are represented as sticks, and color-coding is as follows: pink, WT VP1; orange, E73Q; blue, VQQ; green, E73A.

(D–F) Amino acids 70 to 76 shown as sticks in electron density for variant E73Q (D, orange), E73A (E, green), and VQQ (F, blue).

The figure was created using PyMOL.[22]

entry receptor, HS and CS A/C were added to 293TT cells before and during the infection experiments. Pre-incubation with HS effectively blocked infectious entry of HPV16 and AAV2, which are both known to use GAGs as entry receptors, whereas pre-incubation with CS significantly reduced infectious entry of HPV16 (Figure 5A). In contrast, neither HS nor CS had any effect on WT, VQQ, or N-Q PSV infectivity (Figure 5A). To further confirm that GAGs are not receptors for N-Q variant capsids, 293TT cells were treated with heparinase I/III or chondroitinase ABC to remove HS or CS from the cell surface. Once again, infection by WT, VQQ, and N-Q PSVs was not inhibited by the enzyme treatment, whereas infection by both AAV2 and HPV16 was significantly reduced by heparinase (Figure 5B).

DISCUSSION

In this functional and structural study, we show that mutations in the BC loop of the VP1 protein that occur in KTx recipients impact the ganglioside binding specificity of the BKPyV capsid, and we describe two very different functional patterns, illustrated by the VQQ and N-Q variants.

The N-Q variant has lost the ability to interact with sialic acids due to the mutation of lysine 69 to asparagine. In a previous study, mutation of the same lysine to serine induced a change of tropism from b-series gangliosides to GM1, known to be an SV40 receptor.[3] This emphasizes the essential role that amino acid 69 seems to play in the interaction with gangliosides. Pseudotypes carrying N-Q VP1 had reduced infectivity in both RS and 293TT cells, but the remaining infectivity in 293TT cells was entirely resistant to *C. perfringens* neuraminidase treatment. According to the manufacturer's product description, this enzyme cleaves terminal α2-3, α2-6, and α2-8 linked sialic acid residues, but its activity against glycans with internally branched sialic acid, such as GM2, which was present on 293TT cells, is less clear, raising the possibility that infectivity of the N-Q variant may have involved the GM2 ganglioside. On the other hand, the absence of binding of N-Q VP1 pentamers to GM2 on glycan arrays, the occlusion of the sialic acid binding site by the K69N mutation, and the significantly increased infectivity of N-Q variant PSV in 293TT cells after *C. perfringens* neuraminidase treatment all argue against a role for GM2 and imply that an alternative, sialic acid-independent, receptor is involved in infectious entry of the N-Q variant.

Our first candidates were the HS and CS GAGs, since it has been reported that MCPyV[23] as well as JCPyV and BKPyV pseudotypes can use GAGs for infectious entry.[24] However, in our hands, neither the addition of excess HS or CS nor the removal of HS or CS from 293TT cells had any effect on the infectivity of

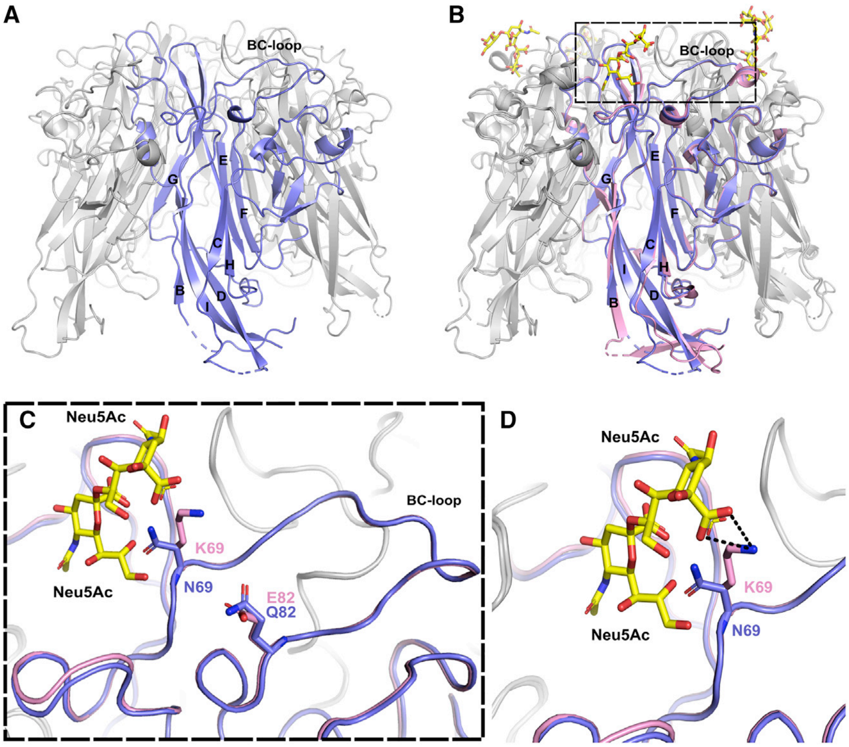

Figure 4. Structure of N-Q VP1 pentamer

(A) Structure of N-Q VP1 pentamer (PDB: 8AH1). VP1 monomer is highlighted in purple.

(B) Superposition of N-Q VP1 pentamer onto WT VP1 pentamers associated with GD3 oligosaccharides (PDB: 4MJ0). WT VP1 monomer is highlighted in pink, and GD3 molecules are represented by yellow sticks.

(C) Zoom on the BC loops of highlighted VP1 monomers. Amino acids 69 and 82 are represented as sticks. Pink, WT VP1; blue, N-Q VP1.

(D) Focus on amino acids in position 69 and their interaction with Neu5Ac. Hydrogen bonds are represented by black dashed lines between K69 and hydroxyl groups of Neu5Ac. The figure was created using PyMOL.[22]

WT, VQQ, or N-Q BKPyV. Our results are not consistent with those of Geoghegan et al.,[24] who reported that incubation with 100 μg/mL heparin resulted in approximately 50% and 75% inhibition of WT BKPyV infection in ART and SFT cell lines, respectively, and complete inhibition of infection by the sialic acid blind BKPyV mutant F76W. Although differences in the cell lines and the particular VP1 mutant used may account for some of the variability between the two studies, another important parameter is the extraction protocol used for PSV preparation. Specifically, we disrupted cells by sonication rather than detergent lysis, and we did not incubate extracts overnight at 37°C before ultracentrifugation, as described in the protocols developed by the Buck lab.[24,25] Although we did not observe major differences in infectious titer in 293TT cells between PSV prepared by the two methods, it is possible that subtle differences in infectivity exist, because the overnight incubation step is thought to be required for capsid maturation by the formation of disulfide bonds between VP1 pentamers in the BKPyV capsid. If capsid maturation is incomplete in our PSVs, it is conceivable that they may not be able to use all the entry pathways that are accessible to replicating viruses. Specifically, they may be able to follow the sialic acid-dependent but not the GAG-dependent infectious entry pathway. This could explain why we did not find GAG-dependent infectivity of BKPyV PSV in any of our assays. We therefore tentatively conclude that an as yet uncharacterized receptor, distinct from GAGs, is involved in the infectious entry of the N-Q BKPyV variant PSVs that we used.

Mutations at VP1 residue 73 had two distinct effects on VP1 structure: first, loss of charge at the capsid surface on the lip of the sialic acid binding site, seen in the E73A, E73Q, and VQQ variants, and secondly, a conformational flip of the BC2 loop with the orientation of amino acids 73 and 74 significantly shifted. Mutation of the initial glutamic acid at position 73 to glutamine in the E73Q variant did not modify the orientation of the BC loop, while its mutation to alanine, in E73A, revealed the flexibility of this part of the BC loop. In the E73A variant crystal structure, two VP1 monomers kept their initial BC loop conformation, two monomers had an alternative orientation of the loop, and the last monomer appeared to have both conformational states of the BC2 loop superposed. In the crystal structure of the VQQ variant, all five monomers showed the alternative, “flipped” conformation. Thus, we conclude that on its own, mutation of amino acid 73 does not induce a stable conformational change of the loop, which requires a second mutation at position 72. The requirement for the A72V mutation in order to stabilize the alternative orientation of BC loop region 73–75 explains the previously reported statistical association of the A72V mutation with E73 mutations in patient samples,[17] and it is consistent with the sequential appearance of VP1 mutations, first at position 73, then followed by A72V, which were seen in patients 3.5 and 3.9 (Figure S1).

Variants E73A, E73Q, and VQQ all showed broader ganglioside binding (Figure 2A), indicating that negative charge at residue 73 may restrict WT BKPyV VP1 binding to b-series gangliosides, and that removing this charge is sufficient to allow binding to a-series gangliosides. On the other hand, only variant E73Q showed strongly enhanced infectivity in RS cells, which as shown by mass spectroscopy, carried the GD1a ganglioside, and only variant E73Q was able to infect LNCaP and GM95 cells supplemented with GD1a. Similarly, in 293TT cells, E73Q retained infectivity equivalent to that of WT VP1, whereas both VQQ and E73A showed significantly reduced infectivity. Therefore, flipping of the BC2 loop, and not loss of negative charge at position 73, was correlated with loss of infectivity in 293TT cells. This led us to a model in which the two different structural effects of VP1 mutations at position 73 have opposite effects on infectivity: loss of charge increases infectivity, by broadening

CellPress
OPEN ACCESS

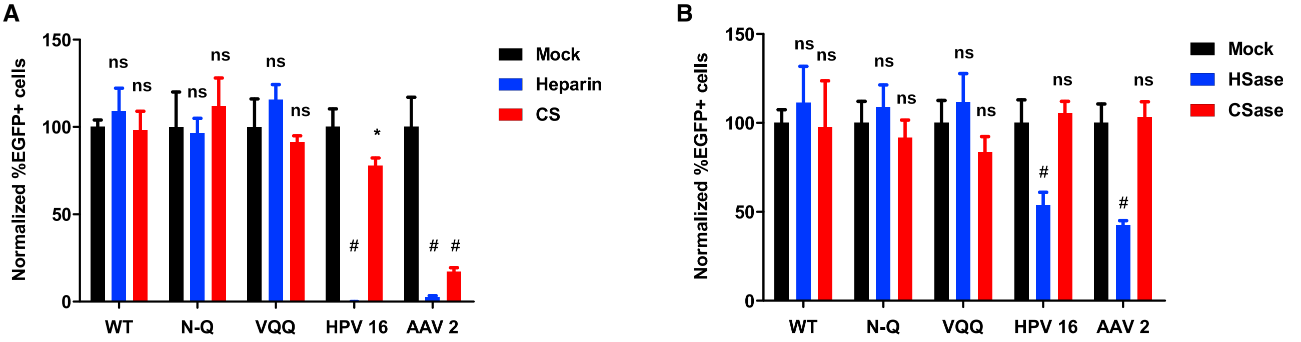

Figure 5. Infectious entry of N-Q and VQQ variant PSV does not involve GAGs

(A) Infectivity assays in 293TT cells in the presence of 100 μg/mL of heparin or chondroitin sulfate A/C with BKPyV WT (2 x 10^6 pEGFP copies/well), VQQ (2 x 10^7 pEGFP copies/well), and N-Q (2 x 10^6 pEGFP copies/well) PSVs. HPV16 (2 x 10^6 pEGFP copies/well) and AAV2 (5 x 10^5 GE/well) PSVs serve as positive control. Error bars correspond to standard deviations (n = 4). *p < 0.05; #p < 0.001; ns, not significant by ANOVA and Dunnet's post-hoc test comparing treatment conditions against infection in medium alone (Mock).

(B) Infectivity assays in 293TT cells treated with heparinase I/III or chondroitinase ABC with BKPyV WT (2 x 10^6 pEGFP copies/well), VQQ (2 x 10^7 pEGFP copies/well), and N-Q (2 x 10^6 pEGFP copies/well) PSVs. HPV16 (2 x 10^6 pEGFP copies/well) and AAV2 (5 x 10^5 GE/well) PSVs serve as positive control. Error bars correspond to standard deviations (n = 4). *p < 0.05; #p < 0.001; ns, not significant by ANOVA and Dunnet's post-hoc test comparing treatment conditions against infection in medium alone (Mock).

ganglioside binding, whereas BC loop flipping reduces infectivity. These two effects explain the infectivity patterns that were observed for the different VP1 variants. In RS cells, the ability to use GD1a as an entry receptor results in the strongly increased infectivity of the E73Q variant, whereas for E73A and VQQ, enhanced ganglioside binding was balanced by a loss of infectivity due to BC loop flipping, leading to near-WT infectivity for both variants. In 293TT cells, although the infectivity of WT, E73Q, and E73A variants was sialic acid dependent, this was not due to gangliosides, since neither a- nor b-series gangliosides were present on 293TT cells (Figure 1C). Therefore, the infectivity of E73Q was not increased relative to WT, and the negative impact of BC loop flipping on the infectivity of E73A and VQQ variants was not compensated by increased ganglioside binding. One previous report described sialic acid-dependent infection by BKPyV that was strongly impacted by tunicamycin, an inhibitor of *N*-linked protein glycosylation, leading the authors to conclude that an *N*-linked glycoprotein, rather than a ganglioside, was an entry receptor for BKPyV.[26] A receptor of this type could be involved in infectious entry in 293TT cells. In the broad-spectrum glycan screening arrays, indeed there was binding with various intensities to sialyl glycans beyond ganglioside sequences (Table S2). These could be followed up further in future structural and functional studies.

Overall, the N-Q and VQQ variants appeared to be almost mirror images of each other. The N-Q variant lost all ganglioside-binding activity but retained infectivity in 293TT cells through a sialic acid-independent pathway, whereas VQQ showed enhanced ganglioside binding but almost completely lost infectivity in 293TT cells. One plausible explanation of these observations is that the VQQ variant may have lost the ability to interact with the unknown entry receptor employed by the N-Q variant to infect 293TT cells, and that this interaction is required, in addition to sialic acid binding, for infectious entry (Figure 6). This would explain why binding and infectivity were uncoupled for the VQQ variant in both 293TT cells and ganglioside-supplemented LNCaP and GM95 cells. Confirmation of this model will require the identification of the putative entry co-receptor, as well as biochemical and structural characterization of its interactions with the BKPyV capsid. One prediction from the structural data presented here is that the WT orientation of the BC loop residues 73–74 will be critical for this interaction.

What are the mutational and selective pressures that drive the emergence of these mutations in patients? As previously noted, the E73Q and E82Q mutations we analyzed occur at optimal APOBEC3A/B editing sites.[18] However, although both the A72V and K69N mutations involve cytosine deamination, they are located in the trinucleotide context G<u>C</u>T, which is not an APOBEC3A/B target site. In addition, the E73A mutation involved a T>G transversion. These observations imply that additional mutagenic processes, independent of APOBEC3A/B, occur in KTx recipients. Once they arise, BC loop mutations conferring neutralization escape appear to be selected by the host humoral response,[17,18] which explains why variants with reduced infectivity relative to WT can rise to dominance in a given individual's viral population. In terms of tissue tropism, although patient 3.4 underwent transplantectomy due to deteriorating graft function at 49 months post-transplant, BKPyV-infected cells could not be clearly identified in kidney tissue at this time (Figure S1A). In contrast, PyVAN was clearly diagnosed in patient 3.9 at a time when the viral population was predominantly WT (Figure S1C), whereas graft function was stable, with no biopsies taken, after the patient's second graft, when the E73A mutation dominated the viral population. Biopsies were not available for patient 3.5 at time points when the VQQ variant was documented, although the graft was still functional, without PyVAN at 70 months post-graft. These observations are consistent with the reduced *in vitro* infectivity of the VQQ and N-Q variants and suggest that BKPyV carrying mutations at K69 and the combination of A72 and E73 mutations had an

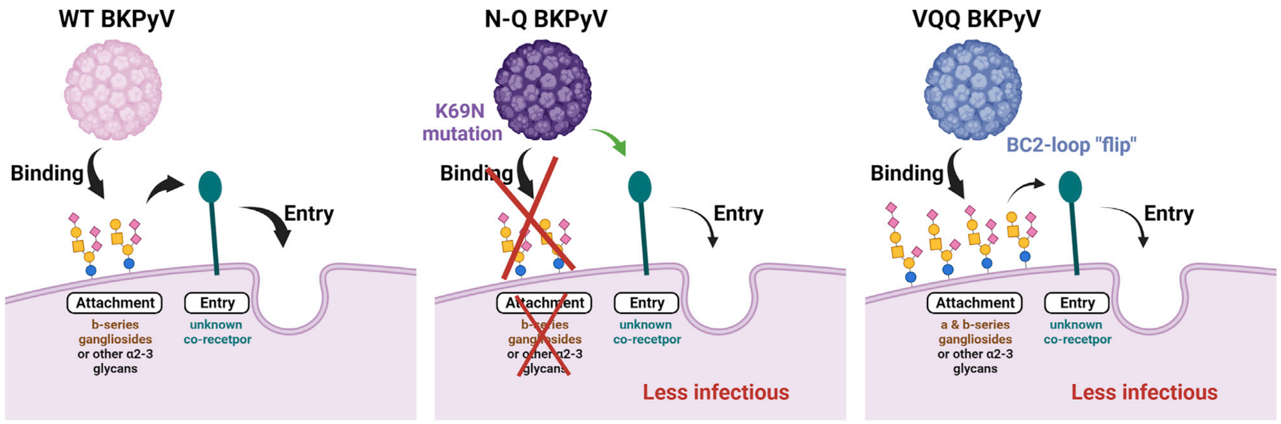

Figure 6. Possible entry mechanisms of N-Q and VQQ variants

This figure illustrates one plausible model for the infectious entry of WT and variant BKPyV analyzed in this study.

We hypothesize that WT BKPyV could use a two-step mechanism for infection involving (1) binding to b-series gangliosides and potentially to α2-3 sialic acid-bearing glycans, followed by (2) interaction with an unknown co-receptor to potentiate entry into cells.

Both the VQQ and N-Q variants had significantly reduced infectivity compared with WT, involving two different mechanisms.

Variant VQQ showed broader ganglioside binding, but this was not sufficient for infectious entry in ganglioside-supplemented cells, clearly indicating the use of a co-receptor for the variant entry into cells. However, the VQQ variant retained some infectivity, suggesting that the interaction with this putative co-receptor was reduced, but not entirely abrogated, by the BC2 loop shift observed in the VQQ VP1 structure.

The N-Q variant lost binding to sialylated glycans, but it retained some infectivity, suggesting that in certain conditions, BKPyV may interact directly with the unknown receptor for entry. The figure was created with BioRender.com.

attenuated phenotype *in vivo*. If this is indeed the case, then the impact of VP1 mutations on pathogenesis in KTx recipients would appear to be very different from that observed in JCPyV infection, where VP1 mutations are clearly associated with progressive multifocal leucoencephalopathy.[27] Confirmation of the benign nature of VP1 mutations in BKPyV infection will require a clinical study to systematically compare graft histology in patients positive for BKPyV DNAemia with and without VP1 mutations. A correlation between the presence of VP1 mutations and the absence of PyVAN could explain why BKPyV DNAemia is not systematically associated with PyVAN[28] and might help clinicians manage the infection. Specifically, the reduction of immunosuppressive therapy may not be necessary for BKPyV with VP1 mutations.

Limitations of the study

Our study has some important limitations. In particular, the description of the structural and functional impact of BKPyV VP1 mutations remains incomplete because additional VP1 mutations accumulated in patients 3.4, 3.5, and 3.9 over time. In patient 3.5, K69M and D75N accrued to the VQQ variant, and in patient 3.4, D60N and A72V were subsequently found in addition to N-Q. However, we were unable to express the $M^{69}V^{72}Q^{73}N^{75}Q^{82}$ and the $N^{60}N^{69}V^{72}Q^{82}$ mutants as either crystallizable VP1 pentamers, VLPs, or high-titer pseudotypes. It was therefore not possible to determine whether subsequent mutations induced more radical changes in virus tropism or, on the contrary, compensated for the deleterious effects on infectivity that we document in the VQQ and N-Q variants. Moreover, we were not able to generate structural data from crystals of the VQQ variant complexed with GD1a ganglioside, so the details of this molecular interaction remain to be determined. Nevertheless, the identification of a naturally occurring "sialic acid blind" VP1 variant does allow us to conclude that a sialic acid-independent entry receptor for BKPyV, relevant for *in vivo* infection, must exist.

STAR★METHODS

Detailed methods are provided in the online version of this paper and include the following:

- KEY RESOURCES TABLE
- RESOURCE AVAILABILITY
  - Lead contact
  - Materials availability
  - Data and code availability
- EXPERIMENTAL MODEL AND SUBJECT DETAILS
  - Patients and clinical samples
  - Cell culture
- METHOD DETAILS
  - Protein expression and purification
  - Crystallisation and structure determination
  - VLP production
  - Pseudovirus production
  - Ganglioside supplementation
  - Enzymatic removal of sialic acid and glycosaminoglycans
  - Inhibition assays with GAGs
  - Glycan array screening
  - Glycolipid extraction and purification
  - Mass spectrometry analysis of GSL
- QUANTIFICATION AND STATISTICAL ANALYSIS

SUPPLEMENTAL INFORMATION

Supplemental information can be found online at https://doi.org/10.1016/j.celrep.2023.112114.

ACKNOWLEDGMENTS

This work was supported by grants from the Agence Nationale de la Recherche (BKNAB ANR-17-CE17-0003), the Université Franco-Allemande (CT-12-20), the German Research Foundation DFG Research Unit FOR2327 ViroCarb, and the Wellcome Trust Biomedical Resource Grants (WT099197/Z/12/Z, 108430/Z/15/Z, and 218304/Z/19/Z). The March of Dimes Prematurity Research Center grant 22-FY18-82 provided partial financial support to the Imperial College Glycosciences Laboratory. The sequence-defined glycan microarrays contain many saccharides provided by collaborators, whom we thank as well as members of the Glycosciences Laboratory for their contribution in the establishment of the neoglycolipid-based microarray system. Y.L. is grateful to Professor Barbara Mulloy and for the excellent facilities provided by Tom Frenkiel and his team (UK MRC Biomedical NMR Center at the Francis Crick Institute) for conducting the NMR analysis of oligosaccharides for the Carbohydrate Microarray Facility over the years, and to Professor Ten Feizi for critical review of the manuscript. For microscopy, we acknowledge the IBISA MicroPICell facility (Biogenouest), member of the national infrastructure France-Bioimaging supported by the Agence Nationale de la Recherche (ANR-10-INBS-04). T.S. is grateful for beam time at the Swiss Light Source (Villigen, Switzerland).

AUTHOR CONTRIBUTIONS

Conceptualization, D.M., F.H., C.B.-B., and T.S.; methodology, W.C.; investigation, M.N.S., A.D.M., L.M.S., D.E., C.P.D., and D.M.; resources, N.-K.N., K.R.-A., and C.B.-B.; writing – original draft, M.N.S., Y.L., and Y.G.; writing – review & editing, D.M.; supervision, Y.G., Y.L., T.S., and D.M.; funding acquisition, Y.L., C.B.-B., and T.S.

DECLARATION OF INTERESTS

The authors declare no competing interests.

Received: August 11, 2022
Revised: December 19, 2022
Accepted: January 30, 2023
Published: February 14, 2023

REFERENCES

1. Shinohara, T., Matsuda, M., Cheng, S.H., Marshall, J., Fujita, M., and Nagashima, K. (1993). BK virus infection of the human urinary tract. J. Med. Virol. *41*, 301–305. https://doi.org/10.1002/jmv.1890410408.
2. Low, J.A., Magnuson, B., Tsai, B., and Imperiale, M.J. (2006). Identification of gangliosides GD1b and GT1b as receptors for BK virus. J. Virol. *80*, 1361–1366. https://doi.org/10.1128/JVI.80.3.1361-1366.2006.
3. Neu, U., Allen, S.A.A., Blaum, B.S., Liu, Y., Frank, M., Palma, A.S., Ströh, L.J., Feizi, T., Peters, T., Atwood, W.J., and Stehle, T. (2013). A structure-guided mutation in the major capsid protein retargets BK polyomavirus. PLoS Pathog. *9*, e1003688. https://doi.org/10.1371/journal.ppat.1003688.
4. Knowles, W.A., Pipkin, P., Andrews, N., Vyse, A., Minor, P., Brown, D.W.G., and Miller, E. (2003). Population-based study of antibody to the human polyomaviruses BKV and JCV and the simian polyomavirus SV40. J. Med. Virol. *71*, 115–123. https://doi.org/10.1002/jmv.10450.
5. Egli, A., Infanti, L., Dumoulin, A., Buser, A., Samaridis, J., Stebler, C., Gosert, R., and Hirsch, H.H. (2009). Prevalence of polyomavirus BK and JC infection and replication in 400 healthy blood donors. J. Infect. Dis. *199*, 837–846. https://doi.org/10.1086/597126.
6. Hirsch, H.H., and Steiger, J. (2003). Polyomavirus BK. Lancet Infect. Dis. *3*, 611–623. https://doi.org/10.1016/S1473-3099(03)00770-9.
7. Hirsch, H.H. (2005). BK virus: opportunity makes a pathogen. Clin. Infect. Dis. *41*, 354–360. https://doi.org/10.1086/431488.
8. Hirsch, H.H., and Randhawa, P.; AST Infectious Diseases Community of Practice (2013). BK polyomavirus in solid organ transplantation: BK polyomavirus in solid organ transplantation. Am. J. Transplant. *13*, 179–188. https://doi.org/10.1111/ajt.12110.
9. Nickeleit, V., Hirsch, H.H., Binet, I.F., Gudat, F., Prince, O., Dalquen, P., Thiel, G., and Mihatsch, M.J. (1999). Polyomavirus infection of renal allograft recipients: from latent infection to manifest disease. J. Am. Soc. Nephrol. *10*, 1080-9.
10. Ramos, E., Drachenberg, C.B., Papadimitriou, J.C., Hamze, O., Fink, J.C., Klassen, D.K., Drachenberg, R.C., Wiland, A., Wali, R., Cangro, C.B., et al. (2002). Clinical course of polyoma virus nephropathy in 67 renal transplant patients. J. Am. Soc. Nephrol. *13*, 2145–2151. https://doi.org/10.1097/01.asn.0000023435.07320.81.
11. Drachenberg, C.B., Hirsch, H.H., Papadimitriou, J.C., Gosert, R., Wali, R.K., Munivenkatappa, R., Nogueira, J., Cangro, C.B., Haririan, A., Mendley, S., and Ramos, E. (2007). Polyomavirus BK versus JC replication and nephropathy in renal transplant recipients: a prospective evaluation. Transplantation *84*, 323–330. https://doi.org/10.1097/01.tp.0000269706.59977.a5.
12. Viscount, H.B., Eid, A.J., Espy, M.J., Griffin, M.D., Thomsen, K.M., Harmsen, W.S., Razonable, R.R., and Smith, T.F. (2007). Polyomavirus polymerase chain reaction as a surrogate marker of polyomavirus-associated nephropathy. Transplantation *84*, 340–345. https://doi.org/10.1097/01.tp.0000275205.41078.51.
13. Babel, N., Volk, H.-D., and Reinke, P. (2011). BK polyomavirus infection and nephropathy: the virus–immune system interplay. Nat. Rev. Nephrol. *7*, 399–406. https://doi.org/10.1038/nrneph.2011.59.
14. Nickeleit, V., Singh, H.K., Randhawa, P., Drachenberg, C.B., Bhatnagar, R., Bracamonte, E., Chang, A., Chon, W.J., Dadhania, D., Davis, V.G., et al. (2018). The Banff working group classification of definitive polyomavirus nephropathy: morphologic definitions and clinical correlations. J. Am. Soc. Nephrol. *29*, 680–693. https://doi.org/10.1681/ASN.2017050477.
15. Nickeleit, V., Singh, H.K., Dadhania, D., Cornea, V., El-Husseini, A., Castellanos, A., Davis, V.G., Waid, T., and Seshan, S.V. (2021). The 2018 Banff Working Group classification of definitive polyomavirus nephropathy: a multicenter validation study in the modern era. Am. J. Transplant. *21*, 669–680. https://doi.org/10.1111/ajt.16189.
16. Randhawa, P.S., Vats, A., Zygmunt, D., Swalsky, P., Scantlebury, V., Shapiro, R., and Finkelstein, S. (2002). Quantitation of viral DNA in renal allograft tissue from patients with BK virus nephropathy. Transplantation *74*, 485–488. https://doi.org/10.1097/00007890-200208270-00009.
17. McIlroy, D., Hönemann, M., Nguyen, N.-K., Barbier, P., Peltier, C., Rodallec, A., Halary, F., Przyrowski, E., Liebert, U., Hourmant, M., and Bressollette-Bodin, C. (2020). Persistent BK polyomavirus viruria is associated with accumulation of VP1 mutations and neutralization escape. Viruses *12*, 824. https://doi.org/10.3390/v12080824.
18. Peretti, A., Geoghegan, E.M., Pastrana, D.V., Smola, S., Feld, P., Sauter, M., Lohse, S., Ramesh, M., Lim, E.S., Wang, D., et al. (2018). Characterization of BK polyomaviruses from kidney transplant recipients suggests a role for APOBEC3 in driving in-host virus evolution. Cell Host Microbe *23*, 628–635.e7. https://doi.org/10.1016/j.chom.2018.04.005.
19. Saito, S., Levery, S.B., Salyan, M.E., Goldberg, R.I., and Hakomori, S. (1994). Common tetrasaccharide epitope NeuAc alpha 2–>3Gal beta 1–>3(Neu-Ac alpha 2–>6)GalNAc, presented by different carrier glycosylceramides or O-linked peptides, is recognized by different antibodies and ligands having distinct specificities. J. Biol. Chem. *269*, 5644–5652.
20. Kawasaki, Y., Ito, A., Withers, D.A., Taima, T., Kakoi, N., Saito, S., and Arai, Y. (2010). Ganglioside DSGb5, preferred ligand for Siglec-7, inhibits NK cell cytotoxicity against renal cell carcinoma cells. Glycobiology *20*, 1373–1379. https://doi.org/10.1093/glycob/cwq116.
21. Hurdiss, D.L., Frank, M., Snowden, J.S., Macdonald, A., and Ranson, N.A. (2018). The structure of an infectious human polyomavirus and its

interactions with cellular receptors. Structure *26*, 839–847.e3. https://doi.org/10.1016/j.str.2018.03.019.

22. Schrödinger, L.L.C. (2015). The PyMOL Molecular Graphics System, Version 1.8.

23. Schowalter, R.M., Pastrana, D.V., and Buck, C.B. (2011). Glycosaminoglycans and sialylated glycans sequentially facilitate merkel cell polyomavirus infectious entry. PLoS Pathog. *7*, e1002161. https://doi.org/10.1371/journal.ppat.1002161.

24. Geoghegan, E.M., Pastrana, D.V., Schowalter, R.M., Ray, U., Gao, W., Ho, M., Pauly, G.T., Sigano, D.M., Kaynor, C., Cahir-McFarland, E., et al. (2017). Infectious entry and neutralization of pathogenic JC polyomaviruses. Cell Rep. *21*, 1169–1179. https://doi.org/10.1016/j.celrep.2017.10.027.

25. Pastrana, D.V., Brennan, D.C., Cuburu, N., Storch, G.A., Viscidi, R.P., Randhawa, P.S., and Buck, C.B. (2012). Neutralization serotyping of BK polyomavirus infection in kidney transplant recipients. PLoS Pathog. *8*, e1002650. https://doi.org/10.1371/journal.ppat.1002650.

26. Dugan, A.S., Eash, S., and Atwood, W.J. (2005). An N-linked glycoprotein with alpha(2,3)-linked sialic acid is a receptor for BK virus. J. Virol. *79*, 14442–14445. https://doi.org/10.1128/jvi.79.22.14442-14445.2005.

27. Gorelik, L., Reid, C., Testa, M., Brickelmaier, M., Bossolasco, S., Pazzi, A., Bestetti, A., Carmillo, P., Wilson, E., McAuliffe, M., et al. (2011). Progressive multifocal leukoencephalopathy (PML) development is associated with mutations in JC virus capsid protein VP1 that change its receptor specificity. J. Infect. Dis. *204*, 103–114. https://doi.org/10.1093/infdis/jir198.

28. Hirsch, H.H., and Randhawa, P.S.; AST Infectious Diseases Community of Practice (2019). BK polyomavirus in solid organ transplantation-guidelines from the American society of transplantation infectious diseases community of practice. Clin. Transplant. *33*, e13528. https://doi.org/10.1111/ctr.13528.

29. Kabsch, W. (2010). XDS. Acta Crystallogr. D Biol. Crystallogr. *66*, 125–132. https://doi.org/10.1107/S0907444909047337.

30. Winn, M.D., Ballard, C.C., Cowtan, K.D., Dodson, E.J., Emsley, P., Evans, P.R., Keegan, R.M., Krissinel, E.B., Leslie, A.G.W., McCoy, A., et al. (2011). Overview of the CCP4 suite and current developments. Acta Crystallogr. D Biol. Crystallogr. *67*, 235–242. https://doi.org/10.1107/S0907444910045749.

31. Liebschner, D., Afonine, P.V., Baker, M.L., Bunkóczi, G., Chen, V.B., Croll, T.I., Hintze, B., Hung, L.W., Jain, S., McCoy, A.J., et al. (2019). Macromolecular structure determination using X-rays, neutrons and electrons: recent developments in Phenix. Acta Crystallogr. D Struct. Biol. *75*, 861–877. https://doi.org/10.1107/S2059798319011471.

32. Emsley, P., Lohkamp, B., Scott, W.G., and Cowtan, K. (2010). Features and development of coot. Acta Crystallogr. D Biol. Crystallogr. *66*, 486–501. https://doi.org/10.1107/S0907444910007493.

33. FlowJoTM Software Version VX (2021). Ashland, OR: Becton, Dickinson and Company.

34. Liu, Y., Childs, R.A., Palma, A.S., Campanero-Rhodes, M.A., Stoll, M.S., Chai, W., and Feizi, T. (2012). Neoglycolipid-based oligosaccharide microarray system: preparation of NGLs and their noncovalent immobilization on nitrocellulose-coated glass slides for microarray analyses. Methods Mol. Biol. *808*, 117–136. https://doi.org/10.1007/978-1-61779-373-8_8.

35. McAllister, N., Liu, Y., Silva, L.M., Lentscher, A.J., Chai, W., Wu, N., Griswold, K.A., Raghunathan, K., Vang, L., Alexander, J., et al. (2020). Chikungunya virus strains from each genetic clade bind sulfated glycosaminoglycans as attachment factors. J. Virol. *94*, e01500-20. https://doi.org/10.1128/JVI.01500-20.

36. Liu, Y., McBride, R., Stoll, M., Palma, A.S., Silva, L., Agravat, S., Aoki-Kinoshita, K.F., Campbell, M.P., Costello, C.E., Dell, A., et al. (2017). The minimum information required for a glycomics experiment (MIRAGE) project: improving the standards for reporting glycan microarray-based data. Glycobiology *27*, 280–284. https://doi.org/10.1093/glycob/cww118.

37. Khan, Z.M., Liu, Y., Neu, U., Gilbert, M., Ehlers, B., Feizi, T., and Stehle, T. (2014). Crystallographic and glycan microarray analysis of human polyomavirus 9 VP1 identifies N-glycolyl neuraminic acid as a receptor candidate. J. Virol. *88*, 6100–6111. https://doi.org/10.1128/JVI.03455-13.

38. Khoo, K.-H., and Yu, S.-Y. (2010). Chapter one - mass spectrometric analysis of sulfated N- and O-glycans. In Methods in Enzymology Glycomics, M. Fukuda, ed. (Academic Press), pp. 3–26. https://doi.org/10.1016/S0076-6879(10)78001–0.

CellPress
OPEN ACCESS

STAR★METHODS

KEY RESOURCES TABLE

REAGENT or RESOURCE	SOURCE	IDENTIFIER
Antibodies		
Anti-SV40 Large T Antigen Antibody, Clone PAb416	Sigma-Aldrich	Cat#MABF121 RRID:AB_2933971
Biological samples		
Kidney biopsies	Biological resource center (CHU Nantes, Hôtel Dieu, Tumorothèque, Nantes, F-44093, France)	N/A
Chemicals, peptides, and recombinant proteins		
Thrombin protease	Cytiva	Cat#27084601
Neuraminidase V from *Clostridium perfringens (C.welchii)*	Sigma-Aldrich	Cat#N2876
Monosialoganglioside GM1 (NH_4^+, salt)	Matreya LLC	Cat#1061
Triganglioside GT1b (NH_4^+, salt)	Matreya LLC	Cat#1063
Disialoganglioside GD1b (NH_4^+, salt)	Matreya LLC	Cat#1501
Disialoganglioside GD1a (NH_4^+, salt)	Matreya LLC	Cat#1062
Trisialoganglioside GT1a	Biorbyt	Cat#orb79737
Disialoganglioside GD3 (NH_4^+, salt)	Matreya LLC	Cat#1504
Heparinase I from *Flavobacterium heparinum*	Sigma-Aldrich	Cat#H2519
Heparinase III from *Flavobacterium heparinum*	Sigma-Aldrich	Cat#H8891
Chondroitinase ABC	Sigma-Aldrich	Cat#C2905
Heparin sodium salf from porcine intestinal mucosa	Sigma-Aldrich	Cat#H4784
Chondroitin sulfate sodium salt from shark cartilage	Sigma-Aldrich	Cat#C4384
DMEM	ThermoFisher Scientific	Cat#11965092
OptiPRO™ SFM	ThermoFisher Scientific	Cat#12309019
RPMI 1640 Medium	ThermoFisher Scientific	Cat#11875093
Fetal Bovine Serum	ThermoFisher Scientific	Cat#26140079
GlutaMAX™ Supplement	ThermoFisher Scientific	Cat#35050061
Penicillin/Streptomycin	Dutscher	Cat#702633
Hygromycin B from *Streptomyces hygroscopicus*	Sigma-Aldrich	Cat#H7772
TrypLE™ Express Enzyme (1X), phenol red	ThermoFisher Scientific	Cat#12605010
Opti-MEM™ I reduced serum medium	ThermoFisher Scientific	Cat#31985062
Triton™ X-100	Sigma-Aldrich	Cat#X100
Pierce™ Universal Nuclease for Cell Lysis	ThermoFisher Scientific	Cat#88701
OptiPrep™ Density Gradient Medium	Sigma-Aldrich	Cat#D1556
Proteinase K	Qiagen	Cat#19131
Hoechst 33,342	Sigma-Aldrich	Cat#382065
Critical commercial assays		
Lipofectamine 2000	Thermo Fisher Scientific	Cat#11668019
Alexa Fluor™ 647 NHS Ester (Succinimidyl Ester)	Thermo Fisher Scientific	Cat#A20006
SYBR™ Green PCR Master Mix	Applied Biosystems	Cat#4309155
Deposited data		
BK Polyomavirus VP1 mutant E73Q	This paper	PDB: 8AGO
BK Polyomavirus VP1 mutant E73A	This paper	PDB: 8AGH
BK Polyomavirus VP1 mutant VQQ	This paper	PDB: 8AH0
BK Polyomavirus VP1 mutant N-Q	This paper	PDB: 8AH1

(*Continued on next page*)

Continued		
REAGENT or RESOURCE	SOURCE	IDENTIFIER
BK Polyomavirus VP1	Neu et al.[3]	PDB: 4MJ1
BK Polyomavirus VP1 in complex with GD3	Neu et al.[3]	PDB: 4MJ0
Experimental models: Cell lines		
Escherichia coli BL21 (DE3)	ThermoFisher Scientific	Cat#EC0114
HEK293 TT cells	National Cancer Institute's Developmental Therapeutics Program	NCI-293TT
RS cells	Evercyte	Cat#CLT-003-0014
GM95 cells	Riken BRC cell bank	Cat#RCB1026
LNCaP cells	Gift from Dr Nathalie Picollet-D'hahan, BIOMICS- LBGE/INSERM U1038<br><br>CEA, Grenoble, France	N/A
Oligonucleotides		
CMV-F 5′-CGCAAATGGGCGGTAGGCGTG-3′	Eurofins Genomics	N/A
pEGFP-N1-R 5′-GTCCAGCTCGACCAGGATG-3′	Eurofins Genomics	N/A
Recombinant DNA		
pET15b-E73Q	BioCat Gmbh	N/A
pET15b-E73A	BioCat Gmbh	N/A
pET15b-VQQ	BioCat Gmbh	N/A
pET15b-N-Q	BioCat Gmbh	N/A
VP2-ph2b	Addgene	Cat#32109
VP3-ph3b	Addgene	Cat#32110
pEGFP-N1	Clontech	Cat#6085-1
pBKV-Ib2	Christopher B. Buck, NCI, USA	Pastrana et al.[25]
pBKV-Ib2-EE	Mutagenesis of pBKV-Ib2	McIlroy et al.[17]
pBKV-Ib2-N-Q	Mutagenesis of pBKV-Ib2	McIlroy et al.[17]
pBKV-Ib2-VQQ	Mutagenesis of pBKV-Ib2	McIlroy et al.[17]
pBKV-Ib2-AE	Mutagenesis of pBKV-Ib2	McIlroy et al.[17]
pBKV-Ib2-QE	Mutagenesis of pBKV-Ib2	McIlroy et al.[17]
p16shell	Addgene	Cat#37320
Software and algorithms		
XDS	Kabsch[29]	https://xds.mr.mpg.de/
CCP4	Winn et al.[30]	https://www.ccp4.ac.uk/
Phenix	Liebschner et al.[31]	https://phenix-online.org/
Coot	Emsley et al.[32]	https://www2.mrc-lmb.cam.ac.uk/personal/pemsley/coot/
PyMOL Molecular Graphics	System Schrodinger, LLC	https://pymol.org/2/
Prism 8	GraphPad	https://www.graphpad.com/scientific-software/prism/
Cellomics Scan software	Thermo Fisher Scientific	N/A
Cellomics View software	Thermo Fisher Scientific	N/A
FlowJo VX	Becton, Dickinson and Company; 2021	https://www.flowjo.com/
Biorender	Biorender	https://biorender.com/
Other		
HisTrap FF Crude 5 mL column	Cytiva	Cat#17528601
HiLoad 16/600 Superdex 200 pg	Cytiva	Cat#28989335
AAV2.CMV.GFP	Translational vector core of the UMR1089 Gene Therapy Laboratory, Nantes	https://umr1089.univ-nantes.fr/en/facilities-cores/cpv

RESOURCE AVAILABILITY

Lead contact

Further information and requests for resources and reagents should be directed to, and will be fulfilled by the lead contact, Dorian McIlroy (dorian.mcilroy@univ-nantes.fr).

Materials availability

All unique/stable reagents generated in this study are available from the lead contact without restriction.

Data and code availability

- Structure data for BKPyV variants: E73Q, E73A, VQQ and N-Q have been deposited in the Protein DataBank and are publicly available as of the date of publication with the accession codes 8AGO, 8AGH, 8AH0 and 8AH1.
- This paper does not report original code.
- Any additional information required to reanalyze the data reported in this paper is available from the lead contact upon request.

EXPERIMENTAL MODEL AND SUBJECT DETAILS

Patients and clinical samples

The three patients in the present study were transplanted between 2011 and 2014, were of male sex, and aged 64, 27, and 56 years. They had previously been included in a prospective observational study approved by the local ethics committee and declared to the French Commission Nationale de l'Informatique et des Libertés (CNIL, n°1,600,141). All patients gave informed consent authorising the use of archived urine samples, blood samples and biopsies for research protocols. Anonymised clinical and biological data for these patients were extracted from the hospital databases. BKPyV VP1 mutations occurring in these patients have been previously described.[17] PyVAN was documented by immunohistochemical staining with mouse monoclonal anti-SV40 T Antigen (Sigma-Aldrich) diluted 1: 50 with polymer-based EnVision FLEX detection system (Dako K8021) utilizing onboard Dako Omnisautomate OMNIS (Dako).

Cell culture

HEK 293TT cells, purchased from the National Cancer Institute's Developmental Therapeutics Program (Frederick, Maryland, USA), were grown in complete DMEM (ThermoFisher) containing 10% FBS (ThermoFisher), 100 U/mL penicillin, 100 μg/mL streptomycin (Dutscher), 1x Glutamax-I (ThermoFisher) and 250 μg/mL Hygromycin B (Sigma-Aldrich). RS cells (Evercyte, Vienna, Austria) were grown in Optipro (ThermoFisher) containing 100 U/mL penicillin, 100 μg/mL streptomycin, 1x Glutamax-I. GM95 cells purchased from the RIKEN BRC cell bank were cultured in DMEM with 10% FBS, 100 U/mL penicillin, 100 μg/mL streptomycin, and 1x Glutamax-I, and LNCaP cells were grown in RPMI medium supplemented with 10% of FBS, 100 U/mL penicillin, 100 μg/mL streptomycin, and 1x Glutamax-I. Cells were maintained at 37°C in a humidified 5% CO_2 incubator, and passaged at confluence by trypsinization for 10 min with 1x TrypLE Express (ThermoFisher).

METHOD DETAILS

Protein expression and purification

VP1 pentamer were produced using plasmid pET15b expression vector encoding BKPyV VP1 mutant amino acid sequences from positions 30–300 with an N-terminal hexahistidine tag (His-tag) and a thrombin cleavage site (BioCat Gmbh). The protein was expressed in E.coli BL21 DE3 by IPTG induction. Proteins were purified first by nickel affinity chromatography using a 5 mL HisTrap FF crude column (Cytiva). After protein sample loading, the column was washed with 20 mM Tris pH 7.5, 250 mM NaCl, 10 mM imidazole and 10% glycerol, and proteins were eluted by applying a gradient of elution buffer composed of 20 mM Tris pH 7.5, 250 mM NaCl, 500 mM imidazole and 10% glycerol. For glycan array analysis, the His-tag was retained while for crystallisation this tag was cleaved with 10 U/mg of thrombin protease (Cytiva) for 24 h at 20°C with agitation. Cleaved and uncleaved pentamers were finally purified by size-exclusion chromatography on a Superdex 200 16/600 column (Cytiva) by eluting protein with 20 mM HEPES pH 7.5 and 150 mM NaCl.

Crystallisation and structure determination

BKPyV VP1 pentamers were concentrated to 3–4 mg/mL and crystallised at 20°C by hanging drop vapor diffusion against reservoir solutions of 10–18% PEG 3.350, 0.1M HEPES pH 7.5, 0.1–0.3 M LiCl (drop size 1 μL of protein/1 μL of reservoir). Crystals were harvested and cryoprotected in reservoir solution supplemented with 20–25% of glycerol for several seconds before flash-freezing them in liquid nitrogen.

Diffraction data were collected at the PXIII beamline of the Swiss Light Source of the Paul Scherrer Institut (Villigen, CH) and processed with XDS.[29] Structures were solved using molecular replacement with Phaser (CCP4)[30] using the wild-type (WT) BKPyV

VP1 structure (PDB: 4MJ1) as a model. Refinement was done using Phenix[31] and the model was built and adjusted in Coot.[32] Structures were deposited in the Protein DataBank with the following accession numbers: E73A mutant - 8AGH; N-Q mutant - 8AH1; E73Q mutant - 8AGO; VQQ mutant - 8AH0. Root-Mean-Square Deviation (r.m.s.d) values were calculated in PyMOL[22] comparing the BC-loop region from amino acids 68 to 76 for variant E73Q, E73A (both conformations) and VQQ to that of the WT structure (PDB: 4MJ1).

VLP production

BKPyV virus-like particles (VLPs) were prepared following the protocols developed by the Buck lab with slight modifications.[25] Briefly, 1.10^7 HEK 293TT cells were seeded in a 75 cm^2 flask in DMEM 10% FBS without antibiotics, then transfected using Lipofectamine 2000 reagent (ThermoFisher) according to manufacturer's instructions. A total of 36 μg VP1 plasmid DNA was mixed with 1.5 mL of Opti-MEM I (ThermoFisher). 72 μL of Lipofectamine 2000 was diluted in 1.5 mL of Opti-MEM I and incubated for 5 min at room temperature prior to mixing with the diluted plasmid DNA. After 20 min at room temperature, 3 mL of DNA-Lipofectamine complexes were added to each flask containing pre-prepared 293TT cells.

Cells were harvested 48h post transfection by trypsinization and washed once in PBS then resuspended in one pellet volume ("v" μL) of PBS, then mixed with 0.4v μL of 25 U/mL type V Neuraminidase (Sigma-Aldrich). After 15 min at 37°C, 0.125v μL of 10% Triton X-100 (Sigma-Aldrich) was added to lyse cells for 15 min at 37°C. The pH of the lysate was adjusted by addition of 0.075v μL of 1M ammonium sulfate, or sodium bicarbonate if VLPs were to be fluorescence-labelled before ultracentrifugation, then 1 μL of 250 U/μL Pierce Nuclease (ThermoFisher) was added to degrade free DNA. After 3h at 37°C, lysates were adjusted to 0.8M NaCl, incubated on ice for 10 min and centrifuged at 5000*g* for 5 min at 4°C. Supernatant was transferred to a new tube and pellet was resuspended in 2 pellet volumes of PBS 0.8M NaCl, then centrifuged. The second supernatant was combined with the first, then pooled supernatant was re-clarified by centrifuging. Cleared lysate from a T75 flask was labeled with 50 μg Alexa Fluor 647 succinimidyl ester (ThermoFisher) for 1 h at room temperature. Labeled or unlabeled lysate was layered onto an Optiprep 27%/33%/39% gradient (Sigma-Aldrich) prepared in DPBS/0.8M NaCl, then centrifuged at 175,000 *g* at 4°C overnight in a Sw55TI rotor (Beckman). For labeled VLPs, visible bands were harvested directly, while for unlabelled VLPs, tubes were punctured with a 25G syringe needle, and ten fractions of each gradient were collected into 1.5 mL microcentrifuge tubes. 6.5 μL of each fraction was kept for SDS-PAGE to verify VP1 purity and determine peak fractions for pooling, then PBS 5% BSA was added to each fraction to a final concentration of 0.1% BSA as a stabilising agent. Peak VP1 fractions were pooled, then the VP1 concentration of each VLP stock was quantified by migrating 5μL on SDS-PAGE, then quantifying the VP1 band by densitometry using a standard curve constructed from a series of 5-fold dilutions of BSA starting at 5 μg/well.

Pseudovirus production

BKPyV and HPV16 pseudovirus (PSV) particles were prepared following the protocols developed by the Buck lab with slight modifications.[25] Briefly, cell preparation and transfection were performed similarly to BKPyV VLP production. However, instead of transfecting only VP1 plasmid, a total of 36 μg plasmid DNA consisting of 16 μg VP1 plasmid, 4 μg ph2b, 8 μg ph3b and 8 μg pEGFP-N1 was transfected into 293TT cells. 48h after transfection, producer cells were collected by trypsinization. The pellet was washed once in cold PBS then resuspended in 800 μL hypotonic lysis buffer containing 25 mM Sodium Citrate pH 6.0, 1 mM $CaCl_2$, 1 mM $MgCl_2$ and 5mM KCl. Cells were subjected to sonication in a Bioruptor Plus device (Diagenode) for 10 min at 4°C with 5 cycles of 1 min ON/ 1 min OFF. Type V neuraminidase was added to a final concentration of 1 U/mL and incubated for 30 min at 37°C. 100 μL of 1M HEPES buffer pH 7.4 (ThermoFisher) was added to neutralise the pH, then 1 μL of 250 U/μL Pierce Nuclease was added before incubation for 2 h at 37°C. The lysate was clarified by centrifuging twice at 5000*g* for 5 min at 4°C and PSV was purified in an Optiprep gradient as described for VLP production. After ultracentrifugation and fraction collection, 8 μL of each fraction was removed for qPCR and the peak fractions were pooled, aliquoted and stored at −80°C for use in neutralisation assays.

For quantification of pEGFP-N1 plasmid, 5μL of each fraction was mixed with 5 μL of proteinase K (stock of 2 mg/mL, Qiagen) and 40 μL of sterile water. This solution was incubated at 55°C for 60 min followed by 95°C for 10 min. Then, 1 μL of the solution was used for qPCR using Applied Biosystems 2x SYBR Green Mix (Applied Biosystems). Primers were CMV-F 5′-CGCAAATGGGCGGT AGGCGTG-3′ and pEGFP-N1-R 5′-GTCCAGCTCGACCAGGATG-3′. Thermal cycling was initiated with a first denaturation step at 95°C for 10 min, followed by 35 cycles of 95°C for 15 s and 55°C for 40 s. Standard curves were constructed using serial dilutions from 10^7 to 10^2 copies of the pEGFP-N1 plasmid per tube.

AAV2-GFP vector was prepared by the Nantes Université CPV core facility (https://sfrsante.univ-nantes.fr/en/technological-facilities/biotherapies/cpv-core-facility).

Ganglioside supplementation

LNCaP cells were seeded at 4.10^5 cells/well in a 12-well Falcon plate for binding experiments and at 10^4 cells/well in 96-well Falcon plate for infectivity experiments.

Gangliosides GM1, GT1b, GD1b, GD1a, GT1a and GD3 (Matreya and Biorbyt) were dissolved in chloroform/methanol/water (2:1:0.1) at 1 mg/mL and stored at −20°C. For supplementation, gangliosides in their storage solution were diluted to desired concentration in RPMI containing 20 mM HEPES, 100 U/mL of penicillin, 100 μg/mL of streptomycin and 1x Glutamax-I. Then, ganglioside solutions were sonicated 4*30s and put in open Eppendorf tubes for 3h at 37°C to allow evaporation of chloroform

and methanol. Ganglioside solution was then added to cells at a final concentration of 5μM and incubated at 37°C for 18h. Culture medium contained 1% FBS during supplementation.

For VLP binding, after ganglioside incorporation, cells were detached with trypsin/EDTA for 15 min at 37°C. Cells were seeded at 25 μL per well in a 96-well V-bottom plate and incubated for 30 min at 4°C with the different BKPyV VLPs coupled with Alexa Fluor 647. After staining, cells were washed with PBS containing 0.5% FBS by centrifuging at 2 000 rpm for 1 min, 3 times. Fluorescence was measured with a Canto II flow cytometer (Becton Dickinson). The flow cytometry results were analyzed using FlowJo VX Software (BD Life Sciences).[33]

For infectivity assays, after ganglioside supplementation, cells were washed twice with complete medium, then PSVs were inoculated. Infection was observed after 5 days by measuring GFP fluorescence with a Cellomics ArrayScan HCS reader (ThermoFisher). The percentage of GFP^+ cells was calculated using Cellomics Scan software with identical settings for all wells analyzed in a single experiment. For visualisation, GFP and Hoechst fluorescence contrast was enhanced across all wells in a plate in Cellomics View software, then images of representative fields exported as.png files.

Enzymatic removal of sialic acid and glycosaminoglycans

293TT cells were seeded at 4.10^5 cells/well in a 12-well Falcon plate for binding experiments and at 10^4 cells/well in 96-well Falcon plate for infectivity experiments. 293TT cells were treated for 1h at 37°C, with 0.5U/mL of Neuraminidase V from *Clostridium perfringens* in DMEM +20 mM HEPES +0.1% BSA or without Neuraminidase V for control conditions. The VLP binding protocol was the same as previously described. For infectivity, cells were inoculated with different PSVs for 3 h and then washed 3 times with complete medium before incubation for 48–72 h at 37°C.

For glycosaminoglycan (GAG) removal, heparinase I/III (Sigma-Aldrich) or chondroitinase ABC (Sigma-Aldrich) were applied to 293TT cells for 2h in digestion medium (20 mM HEPES, pH 7.5, 150 mM NaCl, 4 mM CaCl2 and 0.1% BSA) at 37°C. Diluted PSVs were then added to the wells, and incubated for 3h at 37°C. Enzymes and PSV were then removed, cells were washed 3x in complete medium, then replaced in the incubator in 150μL per well fresh complete medium.

Inhibition assays with GAGs

293TT cells were incubated with 100 μg/mL of heparin (Sigma-Aldrich) or chondroitin sulfate A/C (Sigma-Aldrich), followed by inoculation with PSVs. Infection was observed after 48-72h by measuring GFP fluorescence with Cellomics ArrayScan HCS reader (Micropicell, SFR Bonamy). HPV16 and AAV2 PSVs carrying eGFP plasmid were used as positive controls.

Glycan array screening

The binding specificities of the his-tagged recombinant BKPyV VP1s were analyzed in the neoglycolipid (NGL)-based microarray system.[34] Two versions of microarrays were used: (1) ganglioside-focused arrays featuring 25 glycolipid and NGL probes (Figure 2A), and (2) broad spectrum screening microarrays of 672 sequence-defined lipid-linked glycan probes, of mammalian and non-mammalian type essentially as previously described.[35] The glycan probes included in the screening arrays and their sequences are given in Table S2. Details of the preparation of the glycan probes and the generation of the microarrays are in Supplementary Glycan Microarray Document (Table S3) in accordance with the MIRAGE (Minimum Information Required for A Glycomics Experiment) guidelines for reporting of glycan microarray-based data.[36] The microarray analyses were performed essentially as described.[3,37] In brief, after blocking the slides for 1h with HBS buffer (10 mM HEPES, pH 7.4, 150 mM NaCl) containing 0.33% (w/v) blocker Casein (Pierce), 0.3% (w/v) BSA (Sigma-Aldrich) and 5 mM $CaCl_2$, the microarrays were overlaid with the VP1 proteins for 90 min as protein-antibody complexes that were prepared by preincubating VP1 with mouse monoclonal anti-polyhistidine and biotinylated anti-mouse IgG antibodies (both from Sigma) at a ratio of 4:2:1 (by weight) and diluted in the blocking solution to provide a final VP1 concentration of 150 μg/mL. Binding was detected with Alexa Fluor-647-labelled streptavidin (Molecular Probes) at 1 μg/mL for 30 min. Unless otherwise specified, all steps were carried out at ambient temperature. Microarray imaging and data analysis are described in the Supplementary MIRAGE document (Table S3).

Glycolipid extraction and purification

HEK-293-TT and RS cells were detached from T75 flasks and washed twice with PBS. 5.10^6 cells were lyophilized and extracted twice with $CHCl_3/CH_3OH$ (2:1, v/v) and once with $CHCl_3/CH_3OH$ (1:2, v/v) using intermediary centrifugations at 2500g for 20 min. The combined supernatants were dried under a nitrogen stream, subjected to mild saponification in 0.1 M NaOH in $CHCl_3/CH_3OH$ (1:1, v/v) at 37°C for 2 h and evaporated to dryness. The samples were reconstituted in CH_3OH/0.1% TFA in water (1:1, v/v) and applied to a reverse phase C18 cartridge (Waters, Milford, MA, USA) on a Interchim SPE 6.25ws WorkStation. Reverse phase cartridge was equilibrated in the same solvent. After washing with CH_3OH/0.1% TFA in water (1:1, v/v), GSL were eluted with CH_3OH, $CHCl_3/CH_3OH$ (1:1, v/v) and $CHCl_3/CH_3OH$ (2:1, v/v). The elution fraction was dried under a nitrogen stream prior to structural analysis.

Mass spectrometry analysis of GSL

Isolated glycosphingolipids were permethylated by the sodium hydroxide/DMSO slurry methods[38] at room temperature for 2h under agitation. The derivatization was stopped by addition of water and the permethylated glycans were extracted in $CHCl_3$ and washed at

least seven times with water. Permethylated glycosphingolipids were analyzed by a MALDI-QIT-TOF Shimadzu AXIMA Resonance mass spectrometer (Shimadzu Europe, Manchester, UK) in the positive mode. Samples were prepared by mixing directly on the target 1 μL of glycosphingolipid sample, solubilized in $CHCl_3$, with superDHB matrix solution (10 mg/mL dissolved in $CHCl_3$/CH_3OH (1:1, v/v)) and spotted on the MALDI target. The "mid mode" for a mass range of m/z 1000–3000 was used for scanning and laser power was set to 100, with 2 shots at each of the 200 locations within the circular sample spot.

QUANTIFICATION AND STATISTICAL ANALYSIS

Significant differences seen in the different experiments were calculated through one-way ANOVA followed by Dunnet's test where one condition per experiment was used as a control group; ****$p < 0.0001$, *** (or #) $p < 0.001$, **$p < 0.01$, *$p < 0.05$. All tests were performed using GraphPad Prism 8.

Cell Reports, Volume *42*

Supplemental information

Structural and functional analysis of natural capsid variants suggests sialic acid-independent entry of BK polyomavirus

Marie N. Sorin, Antonio Di Maio, Lisete M. Silva, Domenic Ebert, Clément P. Delannoy, Ngoc-Khanh Nguyen, Yann Guerardel, Wengang Chai, Franck Halary, Karine Renaudin-Autain, Yan Liu, Céline Bressollette-Bodin, Thilo Stehle, and Dorian McIlroy

Patient code	3.4	3.5	3.9
Age at KTx	64	27	56
Sex	M	M	M
Graft type	Kidney 1st graph	Kidney 1st graft	Kidney 2nd graft*
Donor	Deceased	Deceased	Living familial donor
HLA mismatches	3	4	4
Immunosuppressive drug regimen	MMF Tacrolimus	MMF Tacrolimus Corticoids	Azathioprine Tacrolimus
Log10 BKPyV gIb2 neutralizing titre at KTx	2.7	3.3	4.6 (Before KTx2) Serum at KTx1 not available
Log10 BKPyV gIb2 neutralizing titre at M12 post-KTx	4.1	5.1	4.4 (After KTx2)

*The first graft was kidney and pancreas, and ended with PyVAN, requiring transplantectomy followed by a second kidney graft.

Table S1, Related to Results – Patients clinical data

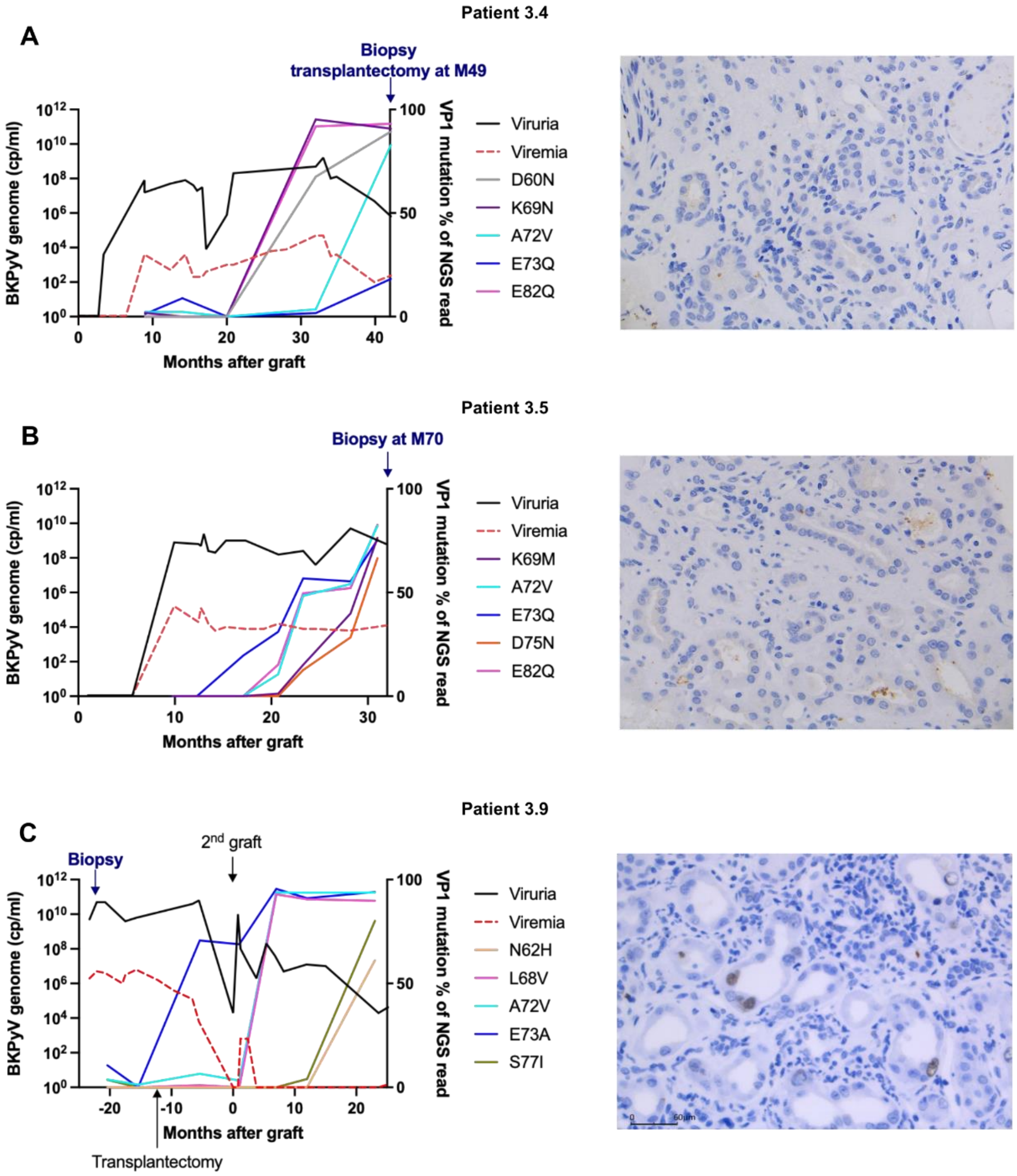

Figure S1, Related to Results. Virological and histological features of BKPyV infection in patients

Left panels: Viruria, DNAemia and VP1 mutation % of NGS reads as a function of time after graft for the three kidney recipients whose VP1 variants were studied. (A) Patient 3.4; (B) Patient 3.5; (C) Patient 3.9. Right panels: SV40 TAg staining of biopsies from patients 3.4 - no significant staining; 3.5 - no significant staining (M70 biopsy is shown); and 3.9 - focal nuclear positivity of tubular epithelial cells. Biopsy images were taken at same magnification. Scale bar is 60 µm.

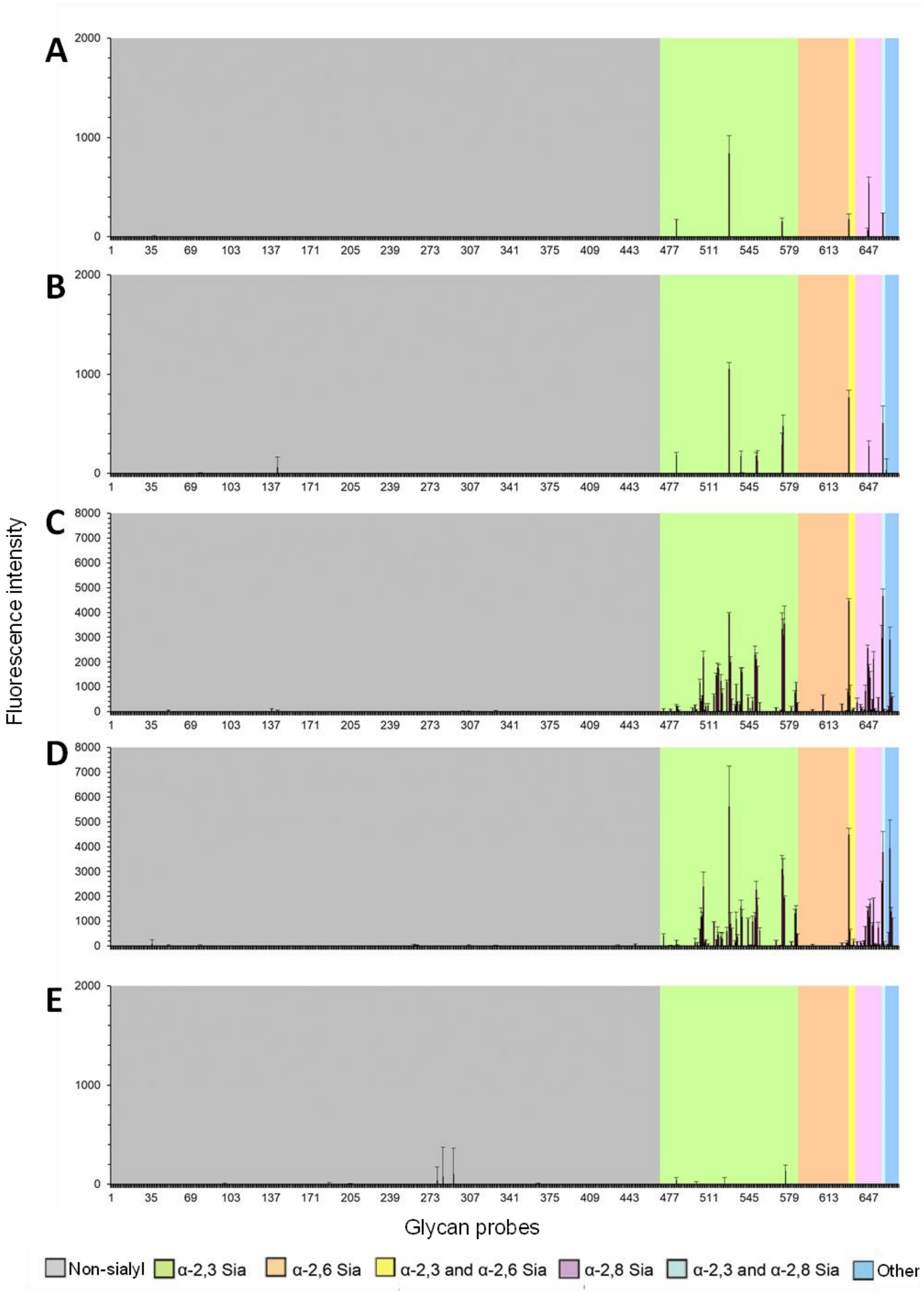

Figure S2, Related to Results. Glycan microarray screening analyses of His-tagged pentameric BKPyV VP1 proteins, wt (A), E73A mutant (B), E73Q mutant (C), VQQ mutant (D) and N-Q mutant (E). The results are the means of fluorescence intensities of duplicate spots, printed at 5 fmol per spot. The error bars represent half of the difference between the two values. In the glycan array the 672 lipid-linked probes are grouped according to sialyl linkages as annotated by the colored panels. The list of glycan probes, their sequences and binding scores are in Table S2.

Classification	Guidelines
**1. Sample: Glycan Binding Sample**	
Description of Sample	Sample names:<br>Wild type (WT) BKPyV VP1<br>Single mutants: E73A BKPyV VP1, E73Q BKPyV VP1<br>Triple mutant: A72V-E73Q-E82Q (VQQ) BKPyV VP1<br>Double mutant: K69N-E82Q (N-Q) BKPyV VP1<br>Origin: recombinant<br>Method of preparation:<br>Please see the "Protein expression and purification" section under *Materials and Methods* in the main text.
Sample modifications	Not relevant.
Assay protocol	Microarray analyses were performed essentially as described [S1], for modifications of the protocol please see "Glycan microarray screening" under *Materials and Methods* section in the main text.
**2. Glycan Library**	
Glycan description for defined glycans	Two glycan microarrays were used, both containing sequence-defined lipid-linked oligosaccharide probes, glycolipids or neoglycolipids (NGLs).<br>1) The 'Ganglioside-focused array' contained 25 ganglioside related probes. These are a sub-set of an array containing 64 glycan probes (in-house designation 'Neuro-Glycan Array Set 1', which will be published elsewhere). The names and sequences of the 25 probes are given in **Figure 2A**.<br>2) A broad-spectrum screening microarray contained 672 sequence-defined oligosaccharide probes **(Table S2)**. These are a sub-set of a recently generated large screening microarray containing around 900 glycan probes (in-house designation 'Array Sets 42-56', which will be published elsewhere).<br>The NGL probes are from the collection assembled in the course of research in the Glycosciences Laboratory (https://glycosciences.med.ic.ac.uk/glycanLibraryList.html).
Glycan description for undefined glycans	Not relevant.
Glycan modifications	No modification was carried out for natural glycolipids.<br>For NGLs, unless otherwise specified these were prepared from reducing oligosaccharides by reductive amination with the amino lipid, 1,2-dihexadecyl-

	sn-glycero-3-phosphoethanolamine [(DHPE) [S2]]; AO, NGLs prepared from reducing oligosaccharides by oxime ligation with an aminooxy functionalized DHPE [(AOPE) [S3]].<br>For full description on the definition of lipid moieties of the glycan probes please see https://glycosciences.med.ic.ac.uk/docs/lipids.pdf.
**3. Printing Surface; e.g., Microarray Slide**	
Description of surface	Nitrocellulose-coated glass microarray slides.
Manufacturer	16-pad UniSart® 3D Microarray Slide from Sartorius (Goettingen, Germany)
Custom preparation of surface	Not relevant.
Non-covalent Immobilisation	The lipid-linked oligosaccharide probes were formulated as liposomes by adding carrier lipids, 1,2-dihexanoyl-*sn*-glycero-3-phosphocholine (DHPC) and cholesterol for arraying and non-covalent immobilization on nitrocellulose-coated glass slides [S1].
**4. Arrayer (Printer)**	
Description of Arrayer	Nano-Plotter 2.1 (GeSiM, Radeberg, Germany).
Dispensing mechanism	Non-contact liquid delivery with four dispensing tips.
Glycan deposition	Approximately 0.33 nl was printed per spot.<br>Lipid-linked glycan probes were printed at 2 and 5 fmol per spot in duplicate.
Printing conditions	The printing solutions were all aqueous based. Printing was performed at ambient temperature and relative humidity of 58%.<br>The 'liposome' printing solutions contained 100 pmol/µl of DHPC and cholesterol (both from SIGMA) as lipid carriers in addition to the lipid-linked glycan probes. The concentrations of the lipid-linked glycan probes were 5 and 15 pmol/µl for the 2 and 5 fmol per spot levels, respectively.<br>The printing solutions also contained Cy3 NHS ester (GE Healthcare) at 20 ng/ml (26 fmol/µl) as a marker to monitor the printing process.
**5. Glycan Microarray with "Map"**	
Array layout	The 'Ganglioside-focused array' was printed in 16-pad format. Each array slide contained 16-pad subarrays. Each pad was set up for printing 64 probes maximum, each at 2 levels in duplicate (four spots for one probe in a row); up to 256 spots (16x16) in total in each pad.<br>The 672 lipid-linked probes in the screening arrays were printed on multiple subarrays for parallel binding analyses.

Glycan identification and quality control	The 'Ganglioside-focused array' was analysed with cholera toxin and a number of polyomavirus VP1 proteins with known specificities, e.g. simian virus 40 VP1 [S4] for quality control purposes.<br><br>The quality control of the screening microarrays of sequence-defined glycan probes was carried out with: biotinylated plant lectins - *Ricinus Communis* Agglutinin I (RCA_{120}), *Aleuria aurantia* lectin (AAL), Concanavalin A (ConA) and WGA (Vector Laboratories), a wide range of anti-carbohydrate antibodies, and a number of viral adhesive proteins that we have published previously.<br><br>These data will be described elsewhere and are available upon request.
**6. Detector and Data Processing**	
Scanning hardware	GenePix 4300A (Molecular Devices, UK)
Scanner settings	Scanning resolution: 10 µm / pixel<br><br>Laser channel: Red (scan wavelength 635 nm)<br><br>PMT: 350<br><br>Scan power: 100% to achieve maximum signal without spot saturation.
Image analysis software	GenePix® Pro 7 (Molecular Devices)
Data processing	The gpr files were entered into an in-house microarray database using software (designed by Mark Stoll, http://www.beilstein-institut.de/en/publications/proceedings/glyco-2009) for data processing. No particular normalization method or statistical analysis was used for the results of the screening arrays.
**7. Glycan Microarray Data Presentation**	
Data presentation	The microarray binding results are in **Figure 2, Figure S2** and **Table S2.**
**8. Interpretation and Conclusion from Microarray Data**	
Data interpretation	No software or algorithms were used to interpret processed data.
Conclusions	The wild type BKPyV VP1 and its E73A, E73Q and VQQ mutants bound to sialylated glycan probes in the arrays with different binding profiles and fluorescence intensities. No significant binding was detected with the N-Q mutant VP1.

Table s3, Related to STAR*METHODS - Supplementary glycan microarray document based on MIRAGE Glycan Microarray guidelines (doi:10.3762/mirage.3).

References:

[S1] Liu, Y., Childs, R. A., Palma, A. S., Campanero-Rhodes, M. A., Stoll, M. S., Chai, W., & Feizi, T. (2012). Neoglycolipid-based oligosaccharide microarray system: preparation of NGLs and their noncovalent immobilization on nitrocellulose-coated glass slides for microarray analyses. Methods in molecular biology (Clifton, N.J.), 808, 117–136. https://doi.org/10.1007/978-1-61779-373-8_8

[S2] Chai, W., Stoll, M. S., Galustian, C., Lawson, A. M., & Feizi, T. (2003). Neoglycolipid technology: deciphering information content of glycome. Methods in enzymology, 362, 160–195. https://doi.org/10.1016/S0076-6879(03)01012-7

[S3] Liu, Y., Feizi, T., Campanero-Rhodes, M. A., Childs, R. A., Zhang, Y., Mulloy, B., Evans, P. G., Osborn, H. M., Otto, D., Crocker, P. R., & Chai, W. (2007). Neoglycolipid probes prepared via oxime ligation for microarray analysis of oligosaccharide-protein interactions. Chemistry & biology, 14(7), 847–859. https://doi.org/10.1016/j.chembiol.2007.06.009

[S4] Campanero-Rhodes, M. A., Smith, A., Chai, W., Sonnino, S., Mauri, L., Childs, R. A., Zhang, Y., Ewers, H., Helenius, A., Imberty, A., & Feizi, T. (2007). N-glycolyl GM1 ganglioside as a receptor for simian virus 40. Journal of virology, 81(23), 12846–12858. https://doi.org/10.1128/JVI.01311-07
